# Membrane expression of the estrogen receptor ERα is required for intercellular communications in the mammary epithelium

**DOI:** 10.1242/dev.182303

**Published:** 2020-03-11

**Authors:** Laurine Gagniac, Mariam Rusidzé, Frederic Boudou, Stephanie Cagnet, Marine Adlanmerini, Pauline Jeannot, Nicolas Gaide, Frank Giton, Arnaud Besson, Ariane Weyl, Pierre Gourdy, Isabelle Raymond-Letron, Jean-Francois Arnal, Cathrin Brisken, Francoise Lenfant

**Affiliations:** 1 INSERM U1048, I2MC, Université de Toulouse, Toulouse 31432, France; 2 Swiss Institute for Experimental Cancer Research, School of Life Sciences, Ecole Polytechnique Fédérale de Lausanne, CH-1015 Lausanne, Switzerland; 3 LBCMCP, Centre de Biologie Intégrative, Université de Toulouse, CNRS, Toulouse 31062, France; 4 LabHPEC Laboratoire d'HistoPathologie Expérimentale et Comparée STROMALab, Université de Toulouse, CNRS ERL5311, EFS, ENVT, Inserm U1031, UPS, Toulouse 31300, France; 5 APHP H.Mondor- IMRB - INSERM U955, Créteil 94010, France

**Keywords:** Estrogen receptor alpha, Membrane initiated signaling, Mammary gland, Stem cells, Paracrine signals, ECM

## Abstract

17β-Estradiol induces the postnatal development of mammary gland and influences breast carcinogenesis by binding to the estrogen receptor ERα. ERα acts as a transcription factor but also elicits rapid signaling through a fraction of ERα expressed at the membrane. Here, we have used the C451A-ERα mouse model mutated for the palmitoylation site to understand how ERα membrane signaling affects mammary gland development. Although the overall structure of physiological mammary gland development is slightly affected, both epithelial fragments and basal cells isolated from C451A-ERα mammary glands failed to grow when engrafted into cleared wild-type fat pads, even in pregnant hosts. Similarly, basal cells purified from hormone-stimulated ovariectomized C451A-ERα mice did not produce normal outgrowths. *Ex vivo*, C451A-ERα basal cells displayed reduced matrix degradation capacities, suggesting altered migration properties. More importantly, C451A-ERα basal cells recovered *in vivo* repopulating ability when co-transplanted with wild-type luminal cells and specifically with ERα-positive luminal cells. Transcriptional profiling identified crucial paracrine luminal-to-basal signals. Altogether, our findings uncover an important role for membrane ERα expression in promoting intercellular communications that are essential for mammary gland development.

## Abstract (precis)

**Summary:** Estrogen receptor (ER) α palmitoylation is required for mammary epithelial cell stem cell function, intercellular and intercompartmental communication with altered expression of paracrine factors and impaired ECM remodeling/protease function.

## INTRODUCTION

Estrogens, particularly 17β-estradiol (E2), are sex hormones that are widely implicated in mammary gland development, which occurs mostly postnatally under endocrine control (Brisken and O'Malley, 2010). E2 binds to two main receptors: the estrogen receptors ERα and ERβ. ERα is required for normal ductal development during puberty (Dupont et al., 2000), while the deletion of ERβ has no effect on postnatal development (Antal et al., 2008). In addition to the crucial role of ERα in mammary gland development, ERα is a key factor in breast cancer diagnosis and treatment. Based on its expression in 70% of breast cancers, hormonotherapy using anti-estrogens, such as tamoxifen and fulvestrant, or aromatase inhibitors are efficacious in reducing recurrence and cancer-related deaths. However, 40% of ER-positive tumors develop resistance and recur. Therefore, studies aiming to identify the mechanisms of ERα action in mammary gland development are important to obtain a better understanding of the genesis of breast cancers.

The mammary gland is composed of an inner luminal layer (luminal cells, LCs) surrounded by an outer layer of myoepithelial/basal cells enriched by mammary stem cells (MaSCs). Basal cells appear ERα negative by immunohistochemistry, and are able to regenerate into basal and LCs in transplantation assays (Visvader and Stingl, 2014; Van Keymeulen et al., 2011) In contrast, ∼30-50% of LCs are ERα positive by immunohistochemistry, most of them co-express the progesterone receptor (PR) (Petersen et al., 1987; Clarke et al., 1997). A second population of LCs shows ER expression at the mRNA level but the protein is not detected by immunohistochemistry (Cagnet et al., 2018). Transplantation of the epithelium from ERα knockout (KO) mice into cleared fat pads revealed a requirement for ERα expression in the epithelium for ductal outgrowth (Mallepell et al., 2006). Moreover, transplantation of a mixture of wild-type and ERα-KO cells induced the proliferation of ERα-deficient cells, showing that E2 exerts its mitogenic effects on the mammary gland through paracrine signaling to promote proliferation and morphogenesis (Mallepell et al., 2006; Brisken and O'Malley, 2010). The expression of amphiregulin, an epidermal growth factor receptor ligand, is highly induced by E2, and this ligand is an important paracrine mediator of estrogen function (Ciarloni et al., 2007). Progesterone receptor (PR) is also an ER target and promotes the expression of strong inducers of mammary development, such as Wnt4 and RANKL, in adulthood (Beleut et al., 2010; Fata et al., 2000; Rajaram et al., 2015). Thus, ERα-positive cells have been dubbed ‘sensor cells’ as they sense the systemic signals and translate them into paracrine cells for neighboring basal and LCs (Pond et al., 2013; Gjorevski and Nelson, 2011).

In response to E2, ERα modulates the transcriptional activity of target genes via its nuclear actions. Over the past two decades, ERα has been shown to associate with plasma membrane caveolae/lipid rafts and to activate non-nuclear signaling, the so-called rapid/non-genomic/membrane initiated steroid signaling (MISS), in a variety of cell types (Arnal et al., 2017; Levin, 2011; Madak-Erdogan et al., 2008). Post-translational modifications, such as palmitoylation, which occurs on cysteine 447 (451 in mice) as part of a nine amino acid motif in the ligand-binding domain of all steroid receptors, has been shown to be crucial for anchoring ERα to the membrane (Acconcia et al., 2005; Pedram et al., 2007). Following association with the heat-shock protein Hsp27, presumably opening up the structure of the receptor, two palmitoyl acyltransferases, DHHC-7 and DHHC-21 (Pedram et al., 2012), attach the palmitoyl acid to the N-terminal Cys of the motif, promoting the physical interaction of ERs with caveolin 1 and its transport to the plasma membrane (Acconcia et al., 2005). The rapid membrane-initiated estrogen signaling indirectly regulates transcription (Madak-Erdogan et al., 2008; Arnal et al., 2017). These MISS effects also act in concert with growth factors, modulating their signaling in certain tissues and cells (Hawsawi et al., 2013; Tian et al., 2012). They appear to play a major role in breast cancer (Levin and Pietras, 2008), and interactions of ERα with Src and PI3K have been observed in aggressive tumors (Poulard et al., 2012). To gain mechanistic insights into the physiological roles of MISS *in vivo*, our laboratory (Adlanmerini et al., 2014) and Levin et al. (Pedram et al., 2014) generated mouse models expressing ERα carrying a mutation of cysteine 451 to alanine, thus abrogating this palmitoylation site and membrane ERα expression (named C451A-ERα and NOER mice, respectively). The C451A-ERα mouse model has revealed a major role for MISS in the vasculature, where it mediates the effects of estrogen on endothelial cells (Adlanmerini et al., 2014). Levin and his collaborators reported that the mammary glands of homozygous NOER female mice completely filled the fat pad but showed diminished ductal side branching and the formation of blunted duct termini (Pedram et al., 2014).

In the present study, we analyze mammary gland development in C451A-ERα mice. There is a transient delay in development during puberty. Intriguingly, C451A-ERα mammary CD24^+^CD29^hi^ cells enriched by MaSCs fail to outgrow in *in vivo* transplantation experiments. This default is rescued by co-injection with wild-type LCs – specifically ERα-positive LCs. Altogether, these data indicate that stem cell properties are not cell intrinsic but rely on intercellular communications that in turn are controlled by the membrane ERα in epithelial mammary cells.

## RESULTS

### C451A-ERα delays pubertal mammary gland development

**Fig. 1.**
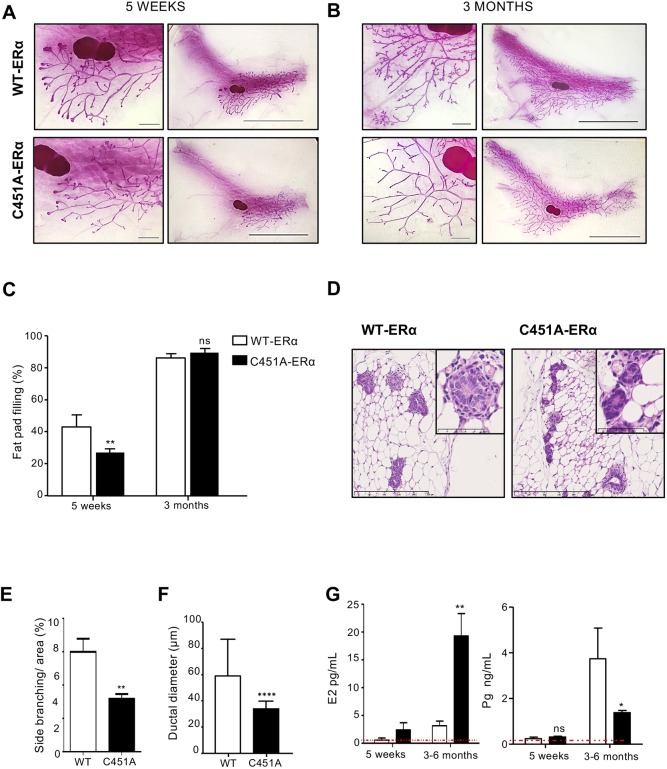
**The invasion of mammary fat pad is delayed at puberty in C451A-ERα mice.** (A,B) Representative images of whole-mount mammary glands from (A) 5-week-old (wild type, *n*=8; C451A-ERα, *n*=8) and (B) 3- to 6-month-old virgin C451A-ERα and wild-type mice (wild type, *n*=9; C451A-ERα, *n*=8). Scale bars: 10 mm (right); 1 mm (left). (C) Quantification of fat pad filling in 5-week-old (two-way ANOVA, *P*<0.01; interaction, **P=0.0082) and 3-month-old (ns, not significant) C451A-ERα and wild-type mice. (D) Representative images of Hematoxylin and Eosin stained transverse sections of mammary glands from 3- to 6-month-old virgin wild-type and C451A-ERα mice. Scale bars: 250 µm and 50 µm (insets). (E,F) Quantification of the number of branching points (E) and of ductal diameters (F) on the ductal tree in 3-month-old wild-type and C451A-ERα mice (wild type, *n*=7; C451A-ERα- *n*=7, *t*-test; **P<0.01; ****P<0.0001). (G) Circulating levels of E2 and progesterone in 5-week- and 3-month-old mice. Expression levels above the dotted line were considered detectable (*P<0.05; **P<0.01; ns, not significant).

To assess the effects of the C451A-ERα germline mutation on mammary gland development, we analyzed mammary glands from C451A-ERα female mice and their wild-type littermates at critical developmental stages. At puberty (5 weeks), fat pad filling was delayed in C451A-ERα females compared with their wild-type littermates (Fig. 1A,C). At the adult stage (3 months), no difference in fat pad filling was observed between the two genotypes (Fig. 1B,C), but C451A-ERα glands showed fewer side branches (Fig. 1E) and thinner ducts observed on transverse sections (Fig. 1D,F), as reported for NOER mice (Pedram et al., 2014).

Histological analysis revealed normal architecture of the ductal tree, attested by the presence of double layer structure by immunohistochemistry with anti-K5 and -K8 cytokeratins (Fig. S1A,B). At 5 weeks of age, when puberty had occurred, C451A-ERα mice presented a significant small decrease of PR-positive cells, with 57.5%±1.1 in the wild type and 51.7±2.1 in the C451A-ERα mice. However, the expression of ERα, the proliferation and the apoptotic rates (Ki-67 and active caspase-3 staining, respectively) were altered neither at puberty nor in adult animals. Western blot analysis of ERα expression confirmed these data (Fig. S1C). Steroid hormone levels were also measured in 5-week-old and 3- to 6-month-old mice. E2 and progesterone levels were comparable in 5-week-old C451A-ERα and wild-type mice; however, in the adult, serum E2 levels were increased in C451A-ERα mice and progesterone levels were substantially decreased compared with their wild-type littermates (Fig. 1G). Altogether, these results indicate a delay in mammary gland outgrowth during puberty, when serum estrogen and progesterone levels are still similar, and a defect in ductal side branching that may be attributable to decreased serum progesterone levels.

### Transplanted C451A-ERα ducts fail to grow in wild-type mice

**Fig. 2.**
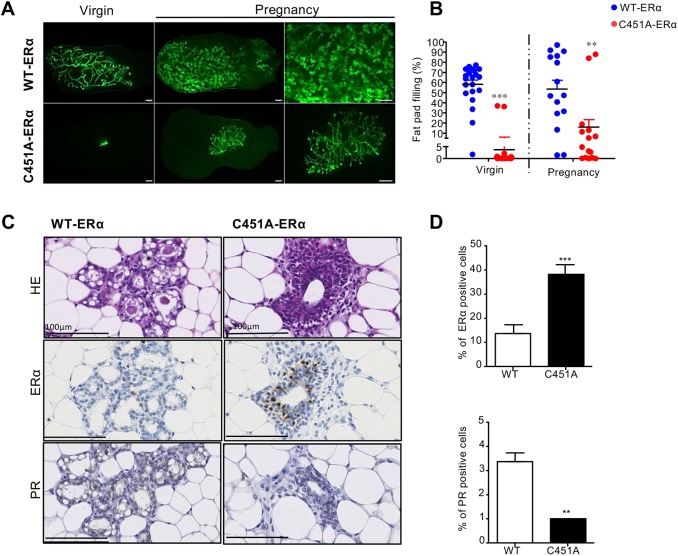
**Absence of development of the C451A-ERα/GFP mammary epithelium after transplantation of ducts in wild-type mice.** (A) Fluorescence stereomicrographs of contralateral inguinal wild-type mammary fat pads engrafted with mammary epithelium from C451A-ERα or wild-type littermates. Images of virgin or day 16-18 pregnant recipients obtained 8 weeks after transplantation. Scale bars: 1 mm. (B) Dot plots showing the extent of fat pad filling by the engrafted epithelia in virgin mice (*n*=22, non-parametric Mann–Whitney *t*-test, ***P<0.001) or pregnant mice (*n*=15, non-parametric Mann–Whitney *t*-test, **P<0.01). Experiments were repeated with four independent donors. Data are mean±s.e.m. (C) Representative immunostaining using Hematoxylin coloration (upper panel), and anti-ERα (middle panel) or anti-PR (lower panel) antibodies in mammary glands from 16.5 day pregnant mice engrafted with epithelium from C451A-ERα or wild-type mice. Scale bars: 100 µm. (D) The proportion of epithelial cells expressing ERα and PR following immunohistochemistry with anti-ERα and PR antibodies was quantified as a percentage of total epithelial cells (**P<0.01; ***P<0.001).

The virgin adult C451A-ERα mice showed altered serum hormone profiles, in particular a substantial decrease in the circulating progesterone level, that might impact the observed alterations in mammary gland morphology (Need et al., 2014). To reveal the epithelial-intrinsic role of the C451A-ERα mutation in mammary gland development, we performed transplantation experiments. These engraftments also allowed us to study the normal development of C451A-ERα mammary glands during regular estrous cycles and pregnancy (alveologenesis), circumventing the infertility of C451A-ERα females (Adlanmerini et al., 2014). A piece of mammary epithelium from C451A-ERα mice was engrafted into a cleared inguinal fat pad from 3-week-old wild-type (*Rag1*^−/−^ or C57BL/6N) mice, whereas the contralateral fat pad was engrafted with wild-type epithelium, as previously described (Mallepell et al., 2006). We used donors that ubiquitously expressed the GFP transgene and visualized the epithelium under a fluorescence stereomicroscope to ensure that comparable amounts of mammary epithelia were engrafted. Eight weeks after surgery, fluorescence stereomicroscopy of grafted glands showed the growth and extension of the wild-type epithelium, and the presence of terminal end buds (TEBs), whereas the C451A-ERα epithelium completely failed to grow (Fig. 2A). A nearly total absence of mammary fat pad filling was observed in more than 22 mice engrafted with the C451A-ERα epithelium (Fig. 2B). C451A-ERα epithelium development remained rudimentary, with, on average, less than 10% of the fat pad filled.

On day 16.5 of pregnancy, when intense hormonal stimulation occurs, alveoli formed all over the ductal tree in the wild-type grafts but not in C451A-ERα epithelia (Fig. 2A), yet the graft expanded (Fig. 2B). Histologically, in wild-type mice, we observed the formation of alveoli lined by a single layer of low columnar epithelial cells, containing lipid droplets (Fig. 2C). Immunofluorescence following the cytokeratins 5 and 8 labeling attested to a double layer structure (Fig. S2A). Expression of ERα and PR by immunochemistry was limited to rare LCs in wild-type mice, as expected with the known decrease of ER-positive cells during pregnancy (Fig. 2C,D) (Van Keymeulen et al., 2017). ERα was significantly expressed in a percentage of LCs while PR was absent in pregnant C451A-ERα mice (a positive control of the PR labeling is presented in Fig. S2B). These observations reveal the importance of palmitoylation of ERα in the mammary epithelium for repopulating the fat pad, and its role in alveologenesis during pregnancy.

### The C451A-ERα mutation alters the balance of luminal/basal mammary epithelial cells and the regenerative potential of MaSCs

**Fig. 3.**
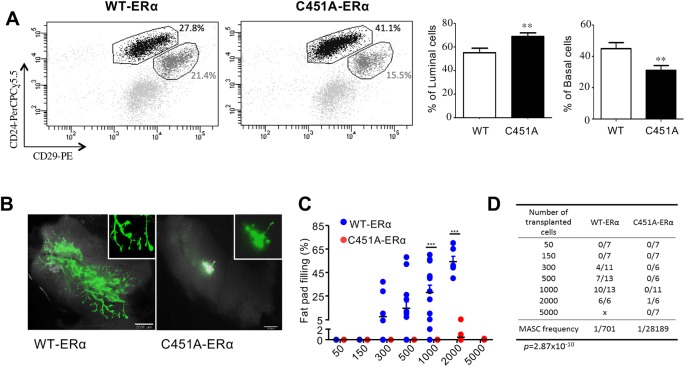
**The frequency and regenerative potential of CD29hiCD24^+^ basal cells are impacted by the C451A-ERα mutation.** (A) Representative flow cytometry dot plots of CD24^+^CD29^lo^ (luminal) and CD24^+^CD29^hi^ (basal) populations gated on CD45 and CD31 from mouse mammary glands obtained from 3-month-old wild-type and C451A-ERα mice. The percentages of luminal or basal cells were calculated within the CD31- and CD45-negative populations (wild type, *n*=25; C451A-ERα, *n*=25, *t*-test, **P<0.01). (B) Representative images of GFP-positive outgrowths arising from transplantation of 2000 double-sorted CD29^hi^ CD24^+^ cells from glands of virgin adult wild-type or C451A-ERα mice. Scale bars: 2 mm. The virgin recipient tissue was collected 8 weeks after transplantation into wild-type and C451A-ERα mice (right panel). (C) Dot plots show the percentages of fat pad filling by outgrowths 8 weeks after transplantation of different numbers of double-sorted CD29^hi^CD24^+^ cells. Cells were injected into the cleared mammary fat pads of 3-week-old syngeneic recipients and collected 8 weeks after transplantation. Data were pooled from two independent experiments (two-way ANOVA, ***P<0.001). Data are mean±s.e.m. (D) Repopulating frequency of the transplantation of limited numbers of double-sorted CD29^hi^CD24^+^ cells from the mammary glands of 12-week-old wild-type or C451A-ERα female mice (ELDA statistical test).

To test the hypothesis that a lack of stem cells may underlie the transplantation defect, we monitored different populations of mammary epithelial cells using flow cytometry. The luminal (CD29^lo^CD24^+^) cell population was increased in C451A-ERα mice, whereas a decrease in the MaSC-enriched (CD29^hi^CD24^+^) subpopulation occurred when these populations were isolated by cell sorting (Fig. 3A). We further investigated whether cell-sorted MaSC from intact C451A-ERα mice were able to repopulate the mammary gland *in vivo*. Transplantation of limited numbers of CD29^hi^CD24^+^ GFP cells from C451A-ERα mice into cleared mammary fat pads revealed an absence of outgrowth compared with similar gate-sorted wild-type cells even when 5000 cells were injected (Fig. 3B,C). Control MaSCs gave rise to extensive outgrowth when at least 300 cells were injected, whereas 98% of outgrowths from MaSCs isolated from C451A-ERα virgin mice filled less than 2% of the fat pad. Only one C451A-ERα mouse presented with 5% outgrowth when 2000 CD29^hi^CD24^+^ cells from mutant C451A-ERα mice were transplanted. The mammary repopulating unit frequency was 1/701 for wild-type cells compared with one in 28,189 for C451A-ERα cells, representing 2.4% of the absolute number of WT mammary repopulating units. Immunohistochemical staining of this small outgrowth using specific anti-cytokeratin K5 and K8 antibodies revealed the presence of both luminal and basal cells (Fig. S3A). Interestingly, ERα immunostaining on this small outgrowth demonstrated that C451A basal cells were able to differentiate into both ERα-positive and ERα-negative LCs, although the ductal elongation was absent (Fig. S3B). Thus, membrane ER is required for the outgrowth of CD29^hi^CD24^+^ cells.

### Engrafted C451A-ERα MaSC cells do not recover their extensive outgrowth ability following hormonal supplementation

**Fig. 4.**
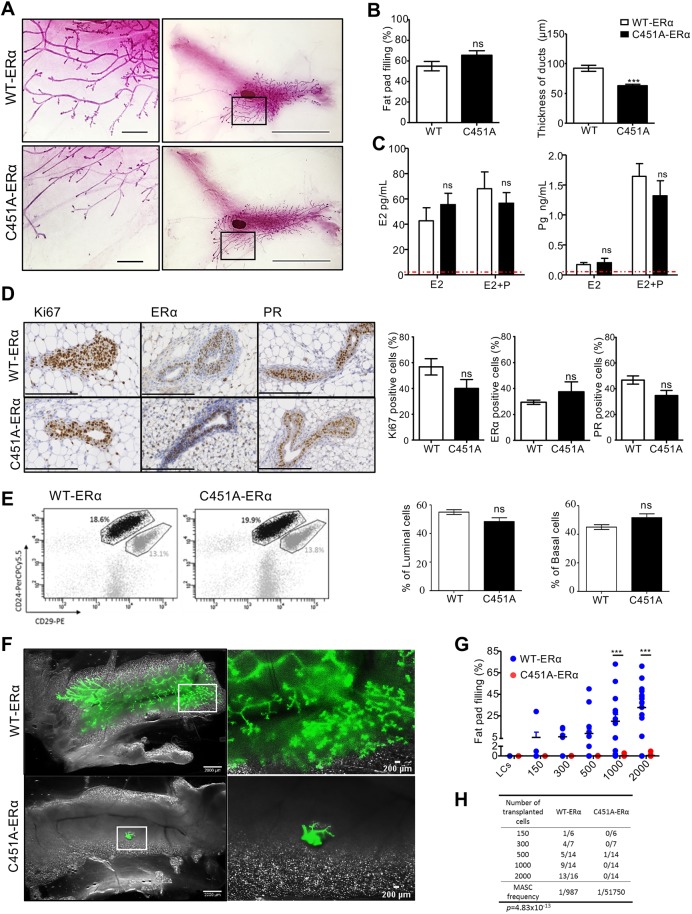
**Hormone supplementation in C451A-ERα mice restores the frequency, but not the regenerative potential, of CD29^hi^CD24^+^ basal cells.** (A) Representative images of whole-mount mammary glands from ovariectomized wild-type and C451A-ERα female mice captured after 3 weeks of treatment with a combination of 17β-estradiol and progesterone; higher magnification images of the ductal tree are also shown. Scale bars: 2 mm (right); 200 µm (left). (B) Bar plots show the percentages of fat pad filling and thickness of ducts (µm) in ductal trees of ovariectomized mice treated with E2 and progesterone for 3 weeks (wild type, *n*=8; C451A-ERα, *n*=11, *t*-test; ns, not significant; ***P<0.001). (C) Circulating levels of E2 and progesterone in ovariectomized mice treated with E2 and progesterone for 3 weeks. Levels above the dotted red line were considered detectable (E2 wild type, *n*=7; E2 C451A-ERα, *n*=5; E2 and Pg wild type, *n*=6; E2 and Pg C451A-ERα, *n*=7; *t*-test; ns, not significant). (D) Representative images of Ki67, ERα and PR immunostaining in mammary glands from ovariectomized wild-type and C451A-ERα mice treated with E2 and progesterone for 3 weeks. The percentages of epithelial cells positive for Ki-67, ERα or PR are expressed relative to the number of total epithelial cells (wild type, *n*=6; C451A-ERα, *n*=6, *t*-test; ns, not significant). Scale bars: 200 μm. (E) Flow cytometry dot plots of CD24^+^CD29^lo^ (luminal) and CD24^+^CD29^hi^ (basal) populations gated on CD45^−^ and CD31^−^ cells from mouse mammary glands removed from ovariectomized wild-type and C451A-ERα mice following 3 weeks of treatment with a combination of 17β-estradiol and progesterone (wild type, *n*=19; C451A-ERα, *n*=21, *t*-test; data not significant). (F) Representative images of GFP-positive outgrowths arising from transplantation of 2000 double-sorted CD29^hi^CD24^+^ cells from the mammary glands of ovariectomized (left panels) wild-type and C451A-ERα (right panel) mice treated with E2+progesterone. Scale bars: 2 mm (left); 200 µm (right). The virgin recipient tissue was collected 8 weeks after transplantation. (G) Percentages of fat pad filling by outgrowths 8 weeks after the transplantation of different numbers of double-sorted CD29^hi^CD24^+^ cells. Cells were injected into the cleared mammary fat pads of 3-week-old syngeneic recipients and collected 8 weeks after transplantation. Data were pooled from two independent experiments (two-way ANOVA, ***P<0.001). (H) Repopulation frequency of the transplantation of a limited number of double-sorted CD29^hi^CD24^+^ cells from the mammary glands of ovariectomized WT and C451A-ERα female mice treated with E2 and progesterone (ELDA statistical test).

Progesterone is responsible for dynamic shifts in specific populations within the mammary epithelial cell hierarchy (Joshi et al., 2010; Asselin-Labat et al., 2010). To investigate whether the shift in cell populations observed in the C451A-ERα mammary glands was secondary to lower progesterone levels, when the hormone has stem cell-promoting effects, mice of both genotypes were ovariectomized at 26 days of age and treated with both E2 and progesterone for 3 weeks. The exposure of C451A-ERα mice to E2 and progesterone did not modify the ability of their mammary ducts to invade the fat pad when compared with their control wild-type littermates (Fig. 4A,B). However, carmine staining revealed an important difference in the architecture of virgin mammary glands. A significant decrease in the thickness of ducts was observed, although this combined treatment efficiently released similar doses of E2 and progesterone in both wild-type and mutant mice (Fig. 4C). According to the immunohistochemistry, obvious changes in the numbers of ERα- and PR-positive cells were not observed (an average of 30-40% positive cells in both and C451A-ERα wild-type mice, Fig. 4D). The proliferation index was not affected. Importantly, hormone treatments restored the balance between luminal (CD29^lo^CD24^+^) and MaSC-enriched basal (CD29^hi^CD24^+^) subpopulations to wild-type ratios (Fig. 4E). Again, we assessed the repopulating MaSC capacities and transplanted limited numbers of CD29^hi^CD24^+^GFP-positive C451A-ERα cells into cleared mammary fat pads and CD29^hi^CD24^+^GFP-positive wild-type cells into the contralateral fat pads. Still, the CD24^+^CD29^hi^ cells from C451A-ERα mice were unable to generate a functional mammary gland, in contrast to wild type (Fig. 4F,G). Ninety-nine percent of outgrowths from MaSC isolated from C451A-ERα virgin mice filled less than 2% of the fat pad, whereas control MaSCs yielded extensive outgrowths in 56% of cases. The mammary repopulating unit frequency was 1 in 987 in ovariectomized wild-type mice supplemented with E2 and progesterone (Fig. 4H), a value that was very similar to intact mice. However, following transplantation with MaSC-enriched basal (CD29^hi^CD24^+^) cells from C451A-ERα, the mammary repopulating unit frequency is approximately 1 in 51,750 for single sorted cells. Thus, inability of C451A-ERα CD24^+^CD29^hi^ cells to reconstitute cleared fat pads is independent of previous intrinsic hormone exposures of stem cells.

### C451A-ERα epithelial cell populations undergo clonal expansion *in vitro* but exhibit defects in matrix degradation

Next, we analyzed the potential of mammary epithelial cells to form colonies (colony-forming cells; CFCs) and mammospheres *in vitro* as a readout for the number of progenitor cells in each population (Stingl et al., 2006). First, FACS-sorted LCs of both genotypes were cultured on irradiated fibroblasts in growth factor-enriched medium. After 8 days, no differences in the number and size of CFCs were observed between C451A-ERα cells and their controls (Fig. S4A). When cells were grown in medium enriched with growth factors containing 4% matrigel, mammospheres were obtained from both sorted luminal and basal cells. We cultured the two subpopulations for more than three generations and did not observe any difference between C451A-ERα and wild-type cells (Fig. S4B). CD24^+^CD29^hi^ cells yielded an average of 300, 200 and 150-200 spheres from 5000 cells seeded at the 1st, 2nd and 3rd generations, respectively. Clonal expansion of the luminal and basal cells was not impacted by the different passages (generations 1 to 3). Having ascertained that clonogenicity is unaffected, we went on to ask whether an inability to invade the mammary stroma may underlie the *in vivo*/*in vitro* discrepancy. We plated equivalent numbers of CD24^+^CD29^hi^ cells onto a fluorescent gelatin matrix *in vitro*. Five days later, the area of degraded gelatin appeared black. The area of degraded gelatin observed with C451A-ERα cells was half of that in wild type (Fig. S5A,B) and was completely abrogated using a nonselective metalloproteinase (MMP) inhibitor (marimastat, Fig. S5C). Degradation was not obtained in experiments using LCs (data not shown). In summary, *in vitro* studies do not reveal a clonogenic difference between populations of wild-type and C451A-ERα luminal and basal epithelial cells. Basal cells harbor outgrowth-matrix interaction defects, suggesting that the inability of C451A-ERα epithelial cells to repopulate fat pads is linked not to the clonogenicity of stem cells but rather to perturbed capacities in establishing interactions with the surrounding tissue *in vivo*.

### Wild-type LCs mediate the expansion of C451A-ERα MaSCs in mixed cell transplantation assays

**Fig. 5.**
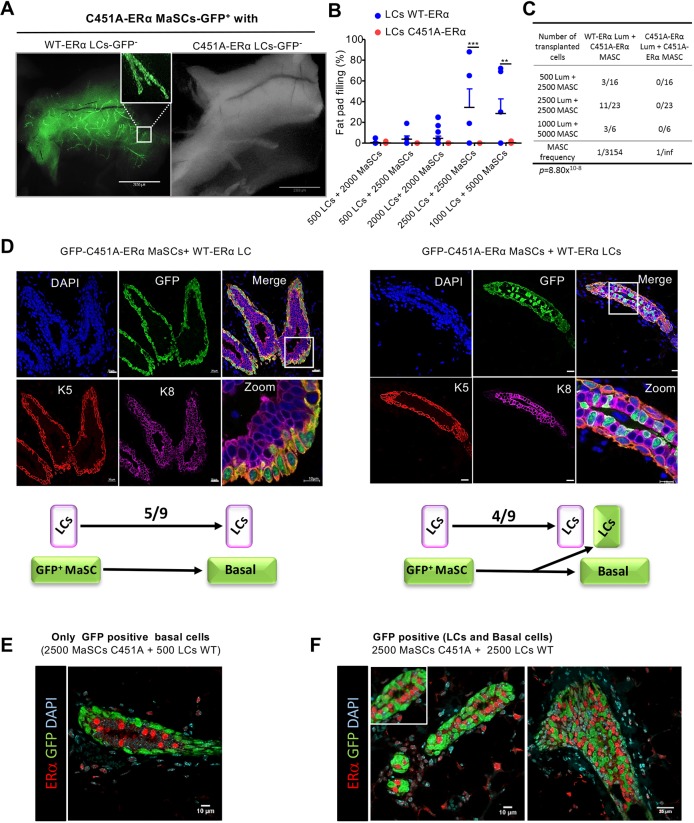
**Co-injection of wild-type CD24^+^CD29^lo^ LCs with CD29^hi^CD24^+^ basal cells from C451A-ERα mice restores their regenerative ability in transplantation assays.** (A) Representative images of GFP-positive outgrowths arising from the transplantation of 2500 double-sorted CD24^+^CD29^lo^ LCs from wild-type (left panel) or C451A-ERα (right panel) mice co-injected with 2500 GFP-positive CD29^hi^CD24^+^ basal cells from C451A-ERα mice. Cells from ovariectomized wild-type or C451A-ERα mice treated with E2+progesterone (Pg) for 3 weeks were sorted by flow cytometry. Scale bar: 2 mm. The wild-type virgin recipient tissue was collected 8 weeks after transplantation. (B) Percentage of fat pad filling by outgrowths 8 weeks after the transplantation of different numbers of double-sorted CD24^+^CD29^lo^ LCs from wild-type or C451A-ERα mice mixed with GFP-positive CD29^hi^CD24^+^ basal cells from C45A-ERα mice. Cells were injected into the cleared mammary fat pads of 3-week-old syngeneic recipients and collected 8 weeks after transplantation. Data were pooled from two independent experiments (two-way ANOVA, **P<0.01, ***P<0.001). (C) Repopulating frequency of the transplantation of limited numbers of double-sorted CD24^+^CD29^lo^ LCs from wild-type or C451A-ERα mice mixed with GFP-positive CD29^hi^CD24^+^ basal cells from C45A-ERα mice (ELDA statistical test). (D) Confocal images of mammary gland epithelium after immunostaining using anti-GFP (green), -K5 (red) and -K8 (magenta) primary antibodies. (E,F) Confocal images of mammary gland epithelium after immunostaining using anti-GFP (green), -ERα (MC20, red) and DAPI (cyan) in epithelium 8 weeks after co-injection of GFP-positive C451A-ERα MaSCs mixed with GFP-negative LCs from wild-type mice. Representative sections of ducts when only C451A MaSCs gave rise to basal cells (E) or when MaSCs C451A gave rise to both basal and luminal GFP positive, but no ERα-positive LCs were observed (F). Scale bars: 2 mm in A; 20 μm in D; 10 µm in E,F (left); 25 µm in F (right).

Abundant literature has demonstrated that some basal cells are multipotent and able to give rise to both luminal and basal lineages upon transplantation (Stingl et al., 2006; Shackleton et al., 2006), while they remain lineage restricted in physiological conditions (Van Keymeulen et al., 2011; Prater et al., 2014; Wuidart et al., 2016). Moreover, paracrine signaling between luminal and basal cells is critically important for mammary epithelial development (Brisken and O'Malley, 2010; Van Keymeulen et al., 2011). To clarify the discrepancy between the normal mammary gland development in hormonally adjusted C451A-ERα females and the absence of outgrowth in reconstitution assays with C451A-ERα basal cells, we transplanted a mixture of FACS-sorted (CD29^hi^CD24^+^) basal cells from C451A-ERα.GFP^+^ mice and GFP-negative CD29^lo^CD24^+^ luminal wild-type or C451A-ERα cells into cleared mammary fat pads of wild-type mice. We used different ratios of C451A basal cells with LCs (1/1 or 5/1) because decreasing the number of LCs has a tendency to preserve the pluripotency of basal cells (Van Keymeulen et al., 2011). Transplantation of GFP-negative wild-type LCs with GFP-positive C451A-ERα basal cells restored their regenerative potential, as extensive outgrowth was observed in 33% of cases (Fig. 5A,B and Fig. S6A). In contrast, transplantation of GFP-positive C451A-ERα mutant basal cells mixed with GFP-negative C451A-ERα LCs failed to regenerate mammary glands. In this transplantation assay, the mammary repopulating unit frequency was 1 in 3154 for single sorted cells (Fig. 5C). This frequency was increased compared with that obtained when C451A-ERα MaSCs were transplanted alone (1 in 28,189 cells), but remained lower than the repopulating frequency unit obtained with WT MaSCs (1 in 701 cells) (Fig. 3D). Analysis of the reconstituted mammary gland under a fluorescence stereomicroscope indicated that the green fluorescent signal was discontinuous (higher magnification image in the left panel of Fig. 5A). Analysis by confocal microscopy of reconstituted mammary glands 8 weeks after transplantation with basal and luminal markers revealed that the vast majority of the basal cytokeratin 5-positive cells were GFP positive in five out of nine mice; these cells originated from the engrafted GFP-positive MaSCs [Fig. 5D (left panel) and Fig. S6B]. Very few LCs were GFP positive. In the other four mice (Fig. 5D, right panel), GFP-positive (K5-positive) basal and (K8-positive) LCs were observed, indicating that the C451A-ERα MaSCs differentiated into LCs in response to paracrine signaling from wild-type LCs. To assess whether the GFP-positive C451A basal cells can give rise to ERα-positive cells when mixed with wild-type LCs, we performed confocal microscopy analysis using anti-GFP and anti-ERα antibodies (Fig. 5E and Fig. S6B-D). We found that the percentage of ERα-positive cells was similar in all the outgrowths (Fig. S6C). However, in the majority of outgrowths, GFP-positive C451A-ERα basal cells gave rise to ERα-negative LCs (Fig. 5E and Movie 1), while GFP and ERα double-positive cells were rarely observed (Fig. S6D). Thus, the C451A-ERα mutation alters the properties of mammary stem cells, as assessed by *in vivo* cell reconstitution assays, possibly owing to the absence of ERα-positive LCs.

### The absence of palmitoylated ERα affects the paracrine signaling of LCs

**Fig. 6.**
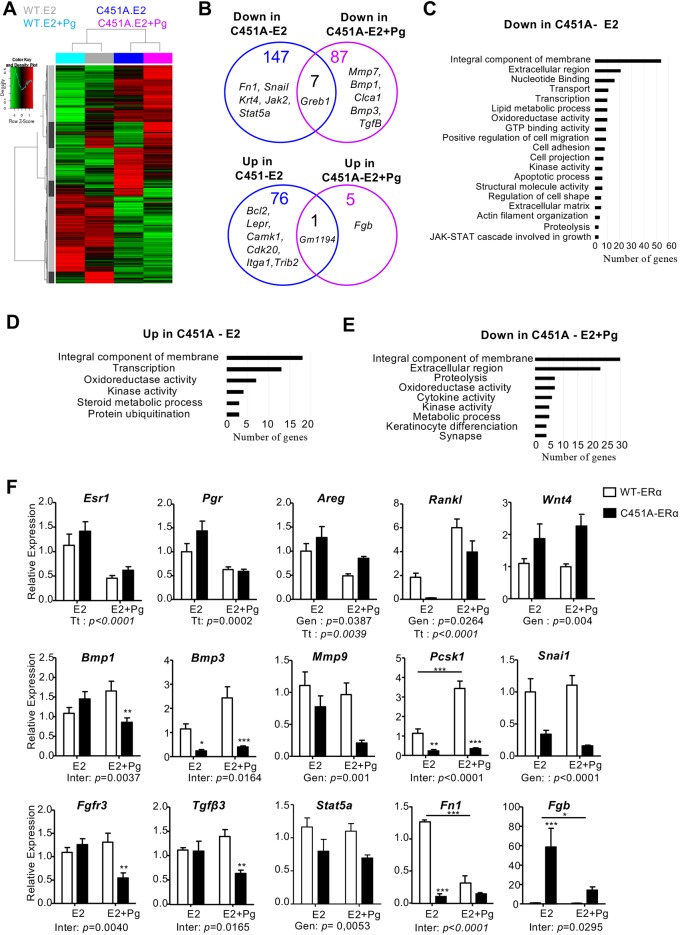
**Large-scale analysis of the effects of C451A-ERα on gene expression in the CD24^+^CD29^lo^ LCs.** (A) Heatmap of the global gene expression analysis in CD24^+^CD29^lo^ LCs from wild-type and C451A-ERα ovariectomized mice treated with E2 or E2+progesterone (Pg) for 3 weeks (wild type, *n*=5; C451A-ERα, *n*=5 in each condition). (B) Venn diagram of differentially expressed (up- and downregulated) genes. (C-E) Gene ontology analysis of the dysregulated genes using the GO database. (F) Gene expression analysis using qRT-PCR. Relative mRNA levels were normalized and presented as relative levels compared with expression in wild-type mice treated with E2. Samples used in the large-scale analysis were included and are complemented with two additional samples prepared using the same conditions (wild type, *n*=7; C451A-ERα, *n*=7; two-way ANOVA, *P<0.05, **P<0.01, ***P<0.001).

To identify transcriptional changes in C451A-ERα LCs that may underlie the observed phenotype (Fig. 5A), we performed a global gene expression analysis on FACS-sorted LCs from ovariectomized C451A-ERα and wild-type mice treated with E2 alone or in combination with progesterone for 3 weeks. Two hundred and thirty one genes were differentially expressed between wild-type and C451A-ERα mice in response to E2 (>1.5-fold, Adj *P*-value <0.05; Fig. 6A,B). The addition of progesterone along with E2 differentially regulated the expression of 100 genes between the two genotypes. More precisely, in response to the progesterone/E2 treatment, only a limited number of genes (six genes, with one gene shared with cells treated with E2 alone) were upregulated in C451A-ERα cells compared with wild-type cells, whereas most genes (94, with seven common genes). Among these downregulated common genes, *Greb1* is one strongly downregulated (fold change of 11 in response to E2; fold change of 17 in response to E2^+^ progesterone) and is well known as an estrogen-responsive gene that is an early response gene in the estrogen receptor-regulated pathway (Fig. 6B). According to gene ontology analysis, most of the differentially expressed genes encode proteins that are integral components of membrane, part of extracellular matrix or display kinase activity (Fig. 6C-E and Fig. S7A).

The results of the microarray analysis were validated by qRT-PCR using Fluidigm Biomark Real-Time PCR. Among the panel of analyzed genes, we also included several genes known to be regulated by ERα and PR in the mammary gland, specifically *Areg* and *Wnt4* (Fig. 6F). *Esr1*, *Pgr* and *Areg* were not differentially expressed in mutant mice, consistent with the immunostaining analyses of ERα and PR protein expression (Fig. 4D). The *RankL* (*Tnfsf11*) gene*,* known to be induced by PR signaling, was indeed upregulated following progesterone treatment, indicating that the progesterone pathway is partially conserved. Interestingly, the *Snai1*, which encodes a zinc-finger transcription factor also known as Snal1 involved in different processes controlling cell differentiation and apoptosis (Côme et al., 2004), was significantly decreased in C451A mice by E2 treatment (Fig. S7A-C). *Fn1*, *Jak2* and *Stat5a* genes were also downregulated by the E2 treatment in C451A-ERα cells. Importantly, a strong gene-gene interaction network was found among these E2-downregulated genes in C451A-ERα cells. These interactions point to genes that positively regulate cell migration (with *Snai1*, *Fn1* and *Lamb1* being the most downregulated genes on this pathway) and involved in the JAK-STAT signaling cascade [a pathway induced by growth-hormone receptors (GH-R) and fibroblast growth factor receptors (FGF-R) (Furth et al., 2011)]. Among the differentially expressed genes observed following addition of progesterone treatment, genes such as *Mmp7*, *Bmp1*, *Bmp3*, *Tgfb1* and *Clca1* (all of which are involved in mammary gland development and extracellular matrix modifications) were downregulated in C451A-ERα mice (Fig. S8A,B). A predicted gene-gene interaction network that is important for duct morphogenesis was found between the morphogens *Bmp1* and *Bmp3*, the *Wnt* signaling pathway (*Dkk3*), and the *Mmp7* metalloprotease. Interestingly, RT-PCR also confirmed downregulation of the growth factor *Tgfb3* or the growth factor receptor *Fgfr3* (which is involved in paracrine signaling of ER-positive LCs). In contrast, the gene whose expression was upregulated to the greatest extent by the progesterone treatment in C451A-ERα mice was *Fgb*, which encodes the extracellular matrix protein fibrinogen and is exclusively expressed in luminal ER-positive cells (Kendrick et al., 2008). Altogether, this gene profile analysis indicated alteration of signaling pathways involving growth factors, extracellular matrix and paracrine signals in the luminal compartment of the C451A-ERα mammary gland.

**Fig. 7.**
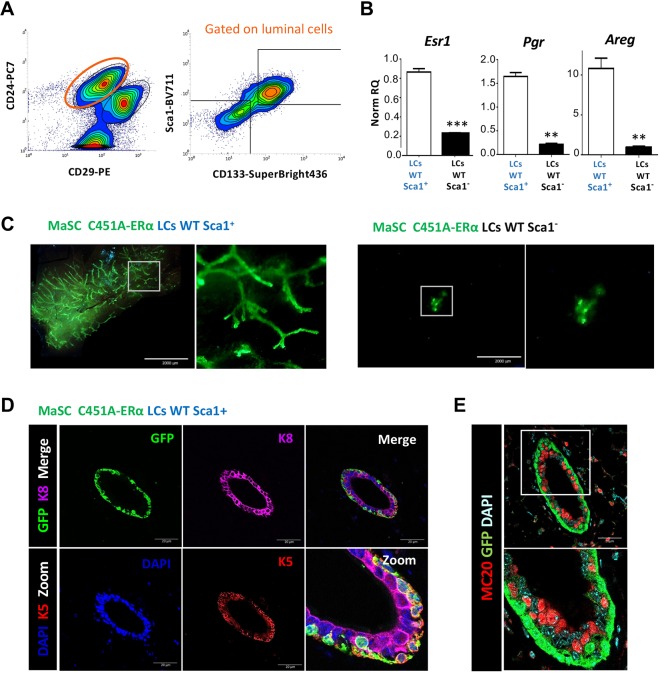
**The wild-type CD24**^**+**^**CD29**^**lo**^ **Sca1**^**+**^ **CD133**^**+**^ **LCs are required for paracrine signaling to restore the regenerative potential of basal cells.** (A) Representative gating strategy illustrating LCs (CD24^+^ CD29^lo^) being subgated for Sca1^+^ CD133^+^ and Sca1^−^ CD133^−^ subpopulations. (B) Relative quantities of *Esr1*, *Pgr* and *Areg* RNA normalized to *B2m*, *Hprt*, *Gusb* and *Tbp* in wild-type Sca1^+^CD133^+^ LCs when compared with wild-type Sca1^−^CD133^−^ LCs (**P<0.01, ***P<0.001; Mann–Whitney test). (C) Representative images of GFP-positive outgrowths arising from the transplantation of C451A-ERα MACS with sorted Sca1^+^ CD133^+^ or Sca1^−^ CD133^−^ wild-type LCs. Scale bars: 2 mm. (D) Confocal images of mammary gland epithelium after immunostaining with anti-GFP (green), anti-K5 (red) and anti-K8 (magenta) primary antibodies. Scale bars: 20 µm. (E) Confocal images of mammary gland epithelium after immunostaining using anti-GFP (green) and anti ERα (red) antibodies. Scale bar: 20 µm.

Finally, in order to analyze which subpopulation of wild-type LCs was important for the signaling to basal cells, we separated by flow cytometry the SCA1^+^/CD133^+^ from the SCA1^−^/CD133^−^ LCs, corresponding (respectively) to the ERα-positive and ERα-negative LCs (identification of which was confirmed by qRT-PCR) (Sleeman et al., 2007; Kendrick et al., 2008; Van Keymeulen et al., 2017) (Fig. 7A,B). This wild-type luminal subpopulation of cells was co-transplanted with C451A-ERα MaSCs at a luminal/basal ratio of 1/5 (see Table S1). Although transplantation of C451A-ERα basal cells mixed with the Sca1^−^/CD133^−^ wild-type LCs cannot reconstitute a normal mammary gland, outgrowths were obtained when C451A-ERa basal cells were mixed with Sca1^+^ CD133^+^ LCs (Fig. 7C). After labeling with anti-GFP and anti-ERα antibodies, GFP C451A-ERα basal cells did not produce detectable double-positive GFP-ERα LCs (Fig. 7D). These additional transplantation assays strongly indicate that there is a failure of mutant ERα-positive LCs at the origin of the phenotype.

## DISCUSSION

Our study of mammary gland development in C451A-ERα mice provides evidence that ERα palmitoylation is important for mammary epithelial cell functions, both for the ability of mammary epithelial cells to establish themselves in the fat pad to promote the outgrowth of ducts, but also for paracrine signaling emanating from ERα positive LCs. Mammary gland development is slightly delayed at puberty, while in adult C451A-ERα mice the mammary gland completely filled the fat pad with decreased side branching. Strikingly, this almost normal *in situ* mammary gland development was substantially different from the total absence of ductal outgrowth observed when C451A-ERα basal cells were transplanted on wild-type stroma, even under the hormonal stimulation of pregnancy. In fact, this dichotomy in the properties of MaSCs between their regenerative potential in transplants and their natural fate under physiological conditions has already been largely described for wild-type MaSCs (Van Keymeulen et al., 2011; van Amerongen et al., 2012; de Visser et al., 2012; Blanpain and Fuchs, 2014). Moumen and their collaborators (2012) have also reported that deletion of the proto-oncogene Myc from the mammary stem cell layer impairs stem cell self-renewal, while not preventing physiological mammary gland fat pad filling. Here, compensatory mechanisms in the germline knockouts from the embryonic C451A-ERα mammary gland likely reconcile these apparently contradictory results.

As C451A-ERα mice exhibited decreased circulating progesterone levels, which has been shown to activate adult MaSC expansion within the mammary cell niche during the reproductive cycle (Joshi et al., 2010; Asselin-Labat et al., 2010), ovariectomized C451A-ERa mice were supplemented with E2 and progesterone. MaSCs from these mice were still unable to repopulate the mammary gland in an intact host *in vivo*. By contrast, no clonogenic difference between both populations of luminal and basal C451A-ERα epithelial cells was observed *in vitro* in CFC assays or in mammosphere cultures. The presence of abundant growth factors in the culture medium used for the *in vitro* clonogenic studies might bypass the missing paracrine signaling required *in vivo*, and might explain this discrepancy. This hypothesis was confirmed by our *in vivo* findings showing that the addition of wild-type LCs to C451A-ERα MaSCs restores their regenerative function by potentially secreting the missing factors. Although it was recently established that the ERα-positive and ERα-negative luminal populations are maintained by lineage-restricted stem cells (Scheele et al., 2017; Dekoninck and Blanpain, 2019; Van Keymeulen et al., 2017), it was shown that ERα-positive cells (sensor cells) are the ones responding to hormone stimulation by sending paracrine signals to the ERα-negative cells to ensure ductal elongation (described as responder cells) (Mallepell et al., 2006; Sternlicht et al., 2006). Our analysis of the rare tiny outgrowths obtained after C451A-ERa MaSC transplantation alone strongly indicated that C45A-ERa MaSCs can give rise to LCs, including ERα-positive LCs. This initial differentiation is also maintained when these MaSCs are mixed with wild-type LCs, but double-positive ERα-GFP cells were very rarely observed. Altogether, these data strongly indicate that the observed phenotype is not due to an inherent failure of MaSCs to perform the initial differentiation, but rather to the alteration of the paracrine signaling from mutated ERα-positive sensing LCs, which prevents their expansion and impairs the function of basal cells required for the final ductal outgrowth. This conclusion is reinforced by results obtained after the engraftment of isolated ERα-positive LCs from wild-type mice.

To try to understand the missing paracrine signaling, we performed large-scale gene analysis in LCs. The transcriptional profiles of the C451A-ERα LCs indicate that the primary responses to hormones were conserved. Indeed, the main factors induced by ERα were not disturbed in C451A-ERα mice; *PgR*, *Areg* and the response to progesterone appear to be preserved because *RankL* expression was increased in C451A-ERα cells treated with E2^+^ progesterone. However, expression of *Greb-1*, well known as an early response gene in the estrogen receptor-regulated pathway is highly affected (Mohammed et al., 2013). Moreover, expression of several major effectors, such as the morphogens BMP1 and BMP3, was substantially decreased by the progesterone treatment in C451A-ERα cells. Importantly, the expression of fibroblast-growth factor receptor FgfR3 was also downregulated. These key paracrine signaling pathways were already described to be required for normal mammary morphogenesis and stem cell function (Sternlicht et al., 2006; Gjorevski and Nelson, 2011; Pond et al., 2013). More precisely, non genomic signaling of ERα was shown to play a pivotal role at puberty in concert with IGF1 to activate the PI3K/Akt pathway (Tian et al., 2012). Expression of some proteases, such as the metalloprotease MMP9 and the serine protease Tmprss6 (matriptase 2), was substantially decreased by the progesterone treatment in C451A-ERα cells, indicating that membrane ERα in LCs is a critical regulator of this paracrine signaling to MaSCs. Indeed, MMPs are ECM-degrading enzymes involved in branching morphogenesis that require epithelial invasion of adipose tissue (Fata et al., 2004). Moreover, a gene network was found in the GO category of genes belonging to the positive regulation of cell migration, involving in particular *Fn1*, *Snai1*, *Jak2* and *Lamb1* (Fig. S7B). Among the genes similarly downregulated by E2 in the C451A-ERα mice, a picture emerges that links membrane ERα signaling in LCs with the *Jak2* and *Stat5a* genes (Fig. S7A-C). Interestingly, the Stat5a signaling pathway is known to be at the crossroad of hormonal and growth factor signaling, which uncovers an important role for membrane ERα in the paracrine signaling of LCs and indicates that membrane ERα is a key regulator of this growth factor sensitivity. Moreover, this Stat5a dysregulation might explain the decreased side branching observed (Furth et al., 2011; Santos et al., 2010). The *Fn1* and *Lamb1* genes, which encode (respectively) fibronectin and laminin B1, two proteins of the extracellular matrix, were also part of the gene network downregulated when membrane ERα was lost (Fig. 6B-F and Fig. S7). Fibronectin was recently described to be involved in the recycling pathway of membrane ERα in MCF7 cells, rescuing ERα from lysosomal degradation, and enhancing its transcriptional activity in response to E2 (Sampayo et al., 2018). The *Snai1* gene in this network is also largely involved in different processes controlling cell differentiation and apoptosis, and acts as a major effector of epithelial cell migration (Côme et al., 2004). Downregulation of this set of genes probably contributes to the observed alterations in the capabilities of basal cells to migrate into gelatin and might also explain the delayed ductal invasion of the fat pad observed at puberty. Overall, these gene pathways and the transplantation experiments of gated ERα-positive wild-type LCs demonstrate that the absence of a phenotype after the transplantation experiment is caused by a failure of mutant ERα-expressing LCs to expand and promote the paracrine signaling for epithelial cell-cell communications and cell-ECM communication. Altogether, these data strongly indicate that the mutation C451A in ERα affects intrinsic properties and paracrine functions of mammary epithelial cells.

An important unanswered question is how do MaSCs sense the initial hormone signals in transplantation experiments? Within MaSCs, Sca1^+^ expression separates ERα-positive cells from ERα-negative cells. Sca1^+^ ER^−^ cells exhibit a higher proliferation rate than Sca1^+^ ER^+^ cells (Dall et al., 2017). Whether these ERα-negative MaSCs cells express membrane ERα at very low levels is difficult to solve, because membrane ERα expression was detectable by immunohistochemistry only in overexpressing conditions in CHO or HEK293 cells (Pedram et al., 2007). Immunodetection of ERα expression in these sorted MaSCs, even in wild type, was unsuccessful (data not shown). In parallel, a single cell analysis recently performed has revealed the presence of a rare basal subset that displays features of mixed-lineage cells that can respond to ovarian hormones and generate luminal progenitors (Pal et al., 2017). However, this subset was not observed in another analysis with settings that are more stringent, arguing that mammary epithelial cells display a differentiation continuum (Bach et al., 2017). Although our microarray analysis was performed using bulk LC analysis, performing single cell RNA profiling on the mammary gland from C451A-ERα mutant mice at different stages of mammary gland development would better refine how absence of membrane ERα affects the differentiation of progenitor cells and the signaling pathways between ERα^+^/ERα^−^ luminal and basal cells.

In conclusion, our study reveals a key role for membrane ERα in the outgrowth abilities of CD24^+^CD29^hi^ cells in transplantation assays, indicating that membrane ERα is required in both luminal and basal cells, particularly for the signaling of ERα-expressing LCs in order to expand and then to activate MaSC in a paracrine manner. Our results provide some mechanistic insights into the nature of the interaction between ERα-negative and ERα-positive epithelial cells that should improve our understanding of the intercellular communication involved in breast development and carcinogenesis.

## MATERIALS AND METHODS

### Mice

The procedures involving experimental animals were performed in accordance with the principles established by the Institut National de la Santé et de la Recherche Médicale (INSERM) and were approved by the local Ethical Committee of Animal Care (CEA-122-DAP-2015-05). The C451A-ERα knock-in mouse line was generated on a C57BL6/N background at the Mouse Clinical Institute as previously described (Adlanmerini et al., 2014). These mice were bred with the C57BL/6 TgN(act-EGFP) GFP-positive mice (Okabe et al., 1997), which were kindly provided by Masaru Okabe (University of Osaka, Japan). Estrous cycle phases were determined in individual adult cycling wild-type and C451A-ERα mice using vaginal cytology (Hennighausen and Robinson, 2005). C451A-ERα mice and corresponding wild-type littermates (WT-ERα) were ovariectomized at 26 days of age. For chronic E2 treatment, ovariectomized mice were implanted with subcutaneous pellets releasing either vehicle or E2 combined with progesterone (P) (0.01 mg/60 days for E2, 1.5 mg/60 days for progesterone; Innovative Research of America).

### Determination of serum hormone levels

Gas chromatography coupled with mass spectrometry (GC-MS) was used to determine serum E2 and progesterone levels using previously described methods (Giton et al., 2015). After clotting, sera were stored at −80°C until hormone assays. E2 levels were determined in two steps.

### Mammary gland whole mounts

Mammary glands whole mounts were generated as previously described (Brisken et al., 1998). Mammary glands of GFP-positive mice were fixed with 4% PFA. Digital images were captured using a Leica Macrofluo microscope equipped with Planapo 1.0× objective. For fluorescent images, an L5 cube (Ex 480/40×, Em 527/30m) was used and images were analyzed using the ImageJ software.

### Immunohistochemistry

Paraffin wax-embedded transverse sections (5 μm) from formalin-fixed mammary gland specimens were stained using anti-Ki-67 (RM-9106, 1/100; Thermo Fisher Scientific), anti-PR (sc-7208, 1/50; Santa Cruz Biotechnology) antibodies or anti active caspase-3 (AF835, 1/800; R&D Systems) as previously described (Abot et al., 2013). For ERα detection (ER-6F11, NCL-L-ER-6F11, Leica), immunohistochemistry was performed with a Dako Autostainer Link 48 on 3 μm sections. Antigen retrieval was performed using a Dako PT Link pressure cooker in pH 6.0 citrate buffer and an EnVision system for antibody detection. Images were acquired using a NanoZoomer Digital Pathology Scanner and NDPView software (Hamamatsu Photonics) for quantification.

### Western blot

Total proteins were separated on 10% SDS/PAGE gels and transferred to nitrocellulose membranes. The following primary antibodies were used: anti-ERα (60C, 04-820, 1/200; Millipore) and anti-GAPDH (sc-32233, dilution; Santa Cruz Biotechnology). Bands were revealed using HRP-conjugated secondary antibodies (HRP-conjugated goat anti-rabbit, 7074S; HRP-conjugated horse anti-mouse, 7076S; Cell Signaling Technology) and visualized through ECL detection, according to the manufacturer's instructions (Amersham Biosciences/GE Healthcare) using a ChemiDoc Imaging System (Bio-Rad). Bands were quantified using densitometry in the ImageJ software.

### Mammary cell preparation

Mammary glands 2, 3, 4 and 5 were dissected from 8- to 12-week-old female mice, and the lymph nodes were removed before processing. After mechanical dissociation into pieces, the tissue was digested in CO_2_-independent culture medium (Gibco) containing 3 mg/ml collagenase A (Roche) and 100 U/ml hyaluronidase (Sigma), supplemented with 5% bovine calf serum for 90 min at 37°C, followed by 0.25% trypsin-EDTA for 1-2 min, 5 mg/ml dispase (Roche) and 0.1 mg/ml DNase (Roche) for 5 min, and 0.64% NH_4_Cl for 3 min. Samples were then filtered through a 40 µm mesh and labeled.

### Cell labeling, flow cytometry and sorting

All labeling steps were performed in PBS supplemented with 2.5% bovine serum albumin (Sigma-Aldrich) and 50 µM EDTA. Cells were first incubated with blocking anti-CD16/CD32 antibodies (14-0161-82, eBioscience) for 10 min at RT before incubation with primary antibodies for 40 min on ice. Primary antibodies included CD24-PerCP-Cy5.5(M1/69, 45-0242, eBioscience), CD29-PE (HMb1, 12-0291, eBioscience), CD31-APC (390, 17-0311, eBioscience), CD45-APC (30-F11, 17-0451, eBioscience), CD133-SuperBright436 (13A4, 62-1331-82, ThermoFisher Scientific) and Sca1-BV711 (D7, 108131, BioLegend). Cells were washed, resuspended in PBS supplemented with 2.5% BSA and 50 µM EDTA before analysis.

Cells were sorted on an INFLUX flow cytometer (BD Bioscience, pressure 20 psi, nozzle 100 µm) using FACS DiVa software. The purity of sorted populations was routinely greater than 95%. Data from live cells, which were initially gated using FACS DiVA software, were analyzed.

### Mammary epithelium transplants

For transplants, the fat pads of 3-week-old *Rag1*^−/−^ or C57BL/6N females were cleared. Pieces of mammary tissue of 1 mm in diameter were prepared from the mammary epithelium of 3-month-old WT-ERα/GFP and C451A-ERα/GFP females under a fluorescence stereomicroscope (Nikon SMZ1500), and inserted into the inguinal prepared fat pads, as previously described (Brisken et al., 1998).

### Mammary epithelial cell transplants

Sorted GFP-positive cells (either GFP-positive basal cells or a mix of GFP-positive basal cells with GFP-negative LCs, as indicated) were resuspended in 10 µl of PBS containing 0.04% Trypan Blue (Sigma) and 50% heat-inactivated fetal calf serum (BWCC), and injected into the inguinal glands of 3-week-old C57BL/6N female mice that had been cleared of endogenous epithelium. Recipient mice were sacrificed 8 weeks after transplantation, unless indicated otherwise. Recipient glands were dissected and analyzed using a Leica Macrofluo microscope with a Planapo 1.0× objective. Outgrowth was defined as an epithelial structure comprising ducts arising from a central point with lobules and/or terminal end buds. For further analysis, some glands were fixed and embedded in paraffin wax for immunostaining. Limiting dilution transplantation assays of basal cells sorted by flow cytometry were performed to determine MaSC functionality and the mammary repopulating unit number *in vivo*. MaSC frequency was calculated at bioinf.wehi.edu.au/software/elda/.

### Confocal microscopy analysis

Paraffin wax-embedded transverse sections (10 μm) from formalin-fixed mammary gland specimens were dewaxed, washed with PBS and subjected to antigen retrieval by boiling in 0.1 M sodium citrate buffer (pH 6) for 20 min and blocking with 2.5% BSA. Cells were fixed with 4% PFA, permeabilized with PBS containing 0.2% Triton X-100 for 3 min, rinsed three times with PBS and blocked with PBS containing 3% BSA, 0.05% Tween20 and 0.08% sodium azide for 20 min before being incubated with primary antibodies diluted in blocking solution for 1 h.

Staining was performed overnight at 4°C with the following primary antibodies: anti-GFP (goat polyclonal, 1/500, ab6673, Abcam), anti-K5 (rabbit Poly19055, 1/500, 905501, BioLegend), anti-K8 (Rat, TROMA-1, 1/7, DSHB), anti-K14 (rabbit, EPR17350, 1/250, ab181595, Abcam) and anti-K18 (rabbit polyclonal, 1/250, ab24561, Abcam). The following secondary antibodies were incubated with the sections for 1 h at room temperature: AlexaFluor 488-conjugated donkey anti-goat (705-545-147, 1/500, Jackson ImmunoResearch), AlexaFluor 594-conjugated donkey anti-rabbit (711-585-152, 1/500, Jackson ImmunoResearch) and AlexaFluor 647-conjugated donkey anti-rat (712-605-153, 1/500, Jackson ImmunoResearch). DAPI was included in the Fluoromount medium. The double staining with anti-GFP (goat poly, 1/500, ab6556, Abcam) and anti-ERα (rabbit polyclonal MC-20, 1/200, Santa Cruz Biotechnology) was performed using the Opal Multiplex IHC kit with the OPAL 520 and OPAL570, respectively, following the manufacturer's recommendations (Perkin Elmer). Sections were imaged using a Zeiss LSM780 confocal microscope. The *z*-series were reconstructed into a 3D movie using the Imaris 9.1.2 software.

### *In vitro* assays

Freshly sorted LCs (1000 cells) were resuspended in culture medium [DMEM/F12 lacking Phenol Red supplemented with 5 µg/ml insulin (Sigma), 10 ng/ml EGF (Sigma), 100 ng/ml cholera toxin (Sigma) and 5% FCS] and seeded onto 24-well plates in the presence of 5000 irradiated NIH-3T3 cells, as previously described (Sleeman et al., 2007). Five days later, colonies were fixed with 4% PFA, stained with Hematoxylin and Eosin, and counted.

For three-dimensional mammosphere assays, FACS-sorted luminal or basal cells (10,000 cells) were resuspended in culture medium [DMEM-F12 lacking phenol red supplemented with B27 (1×, Gibco), 20 ng/ml EGF (Sigma), 20 ng/ml bFGF (Gibco), 4 µg/ml heparin (Sigma), 10 µg/ml insulin (Sigma) containing 4% Matrigel] as previously described (Dontu et al., 2003; Spike et al., 2012). After 15 days in culture, mammospheres were imaged using a stereomicroscope (Nikon SZM800). Three independent experiments were analyzed. For each traced organoid, the size and number of clones were measured using ImageJ software (NIH). For serial passaging, mammospheres were collected by centrifugation and incubated with 0.05% trypsin/EDTA (Gibco) to obtain a single cell suspension. Cells were replated in 4% Matrigel (BD Pharmingen) at a density of 5000 cells/ml, as described above. All cultures were maintained in a 5% CO_2_ atmosphere at 37°C.

### Gelatin degradation assay

Coverslips were cleaned overnight in 1 M HCl, washed four times with ddH_2_O and then successively coated with 50 μg/ml poly-L-lysine, 0.5% glutaraldehyde, fluorescent gelatin (1:10 mixture of Oregon green 488-conjugated gelatin from pig skin (G13186, Molecular Probes) and 0.2% gelatin from bovine skin (Sigma G1393), and 5 mg/ml sodium borohydride (Sirmans et al., 2014). Between each coating, coverslips were washed three times with PBS. Coverslips were then sterilized with 70% ethanol and 30,000 basal cells were seeded and incubated for 5 days. When mentioned, medium was supplemented with marimastat (a non selective MMP inhibitor, BB25.16, Euromedex, 5 µM). Culture medium and drugs were replaced every two days, cells were fixed with 4% PFA and stained.

### Microarray analysis

Luminal cells (four samples for wild-type LCs and five for the other conditions) were sorted into 0.04 M RLT-DTT medium (Qiagen) and stored at −20°C. A Qiagen RNeasy micro-kit (Qiagen) was used to extract mRNAs. Gene expression profiles were analyzed at the GeT-TRiX facility (GenoToul) using Agilent Sureprint G3 Mouse GE V2 microarrays (8×60K, design 074809) according to the manufacturer's instructions. For each sample, cyanine 3 (Cy3)-labeled cRNAs were prepared from 25 ng of total RNA using the One-Color Quick Amp Labeling kit (Agilent) according to the manufacturer's instructions, followed by Agencourt RNAClean XP (Agencourt Bioscience Corporation). Dye incorporation and cRNA yield were examined using a Dropsense 96 UV/VIS droplet reader (Trinean). Cy3-labeled cRNAs (600 ng) were hybridized on the microarray slides according to the manufacturer's instructions. Immediately after washing, slides were scanned on an Agilent G2505C Microarray Scanner using Agilent Scan Control A.8.5.1 software, and fluorescence signals were extracted using Agilent Feature Extraction software v10.10.1.1 with the default parameters.

Microarray data were analyzed using R (R Development Core Team; http://www.R-project.org) and Bioconductor packages (www.bioconductor.org, v3.0; Gentleman et al., 2004) as described in GEO deposit GSE142297). Raw data (median signal intensity) were filtered, log2 transformed, corrected for batch effects (microarray washing bath and serial labeling) and normalized using the quantile method (Bolstad et al., 2003). The list of selected genes was established from microarray analyses with a fold change <1.5 or >1.5 and an adjusted *P*-value <0.05. Functional analyses were performed using DAVID Bioinformatics Resources 6.7 (david.abcc.ncifcrf.gov) and comparisons were achieved with the Venn Diagrams plug-in based upon the VENNY tool developed by J. C. Oliveros (https://bioinfogp.cnb.csic.es/tools/venny/index.html).

### Gene expression analysis using qRT-PCR

For luminal gene expression profiling, we performed a quantitative PCR (Fluidigm Dynamic Array, Fluidigm platform, GeT facility, GenoToul) on a set of 37 genes selected from the microarray data and a literature search. Primers were validated by testing PCR efficiency using standard curves (95%<efficiency<105%). Gene expression was quantified using the comparative Ct (threshold cycle) method. HPRT1, β2M and GUSb were used as reference genes.

### Statistics

Statistical analyses were performed using Prism 5 software (GraphPad). Data are presented as mean±s.e.m. Comparisons between two specific groups were performed using Student's *t*-test. To test the effect of treatments or genotypes, data were compared between multiple groups with one variable a using one-way ANOVA followed by a Mann–Whitney post-hoc multiple comparison test. To test the interaction between treatments and genotypes, a two-way ANOVA was used, followed by the Bonferroni's post-hoc test when an interaction was observed. *P*<0.05 was considered statistically significant (*P<0.05; **P<0.01; ***P<0.001; ns, not significant).

## Supplementary Material

Supplementary information
