# Supplementary material for: Membrane expression of the estrogen receptor ERα is required for intercellular communications in the mammary epithelium
Source: Development. 2020 Mar 11;147(5):dev182303. doi: 10.1242/dev.182303 (PMC7075076; doi:10.1242/dev.182303)
Supplement: Supplementary information [file develop-147-182303-s1.pdf]

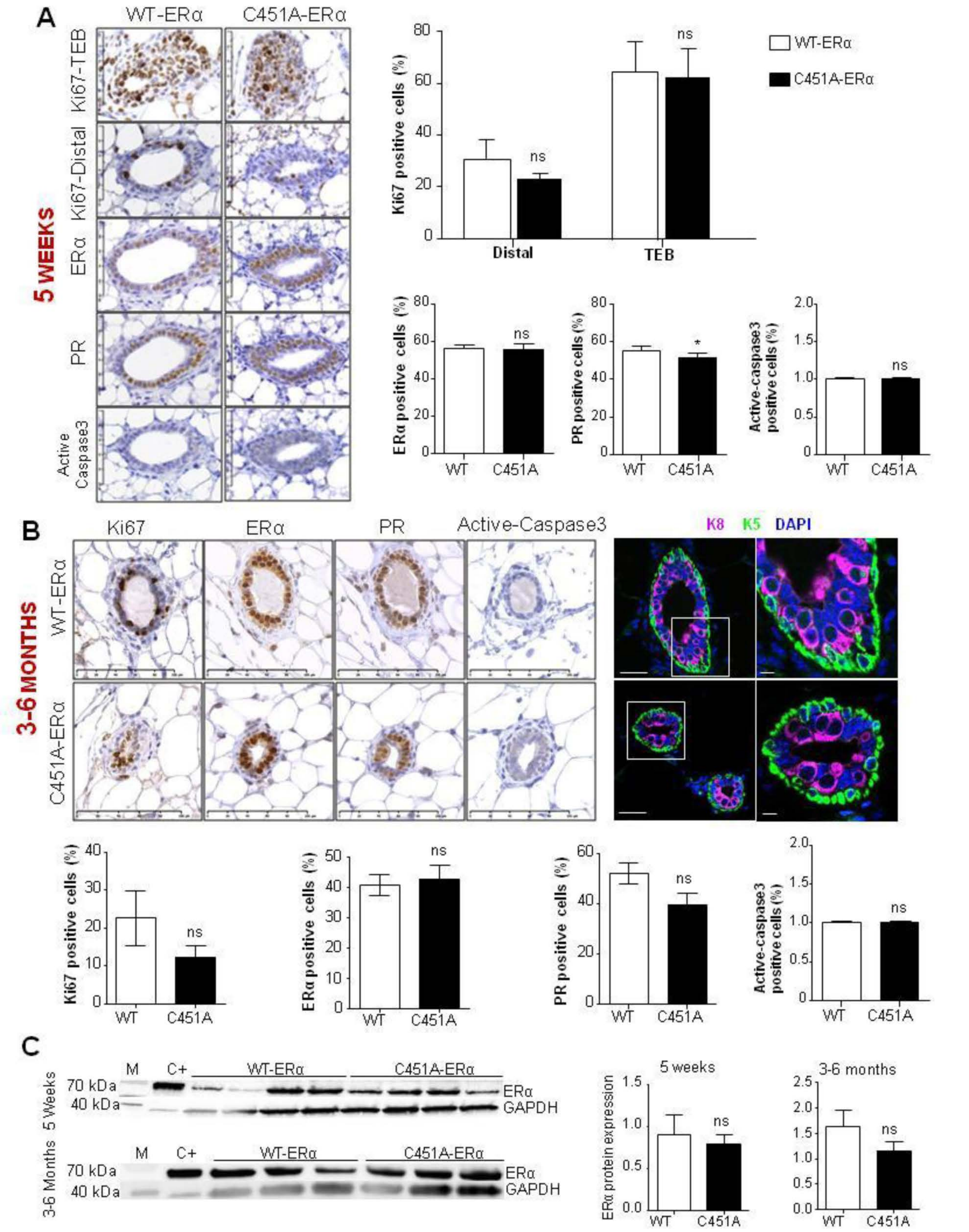

Development • Supplementary information

Figure S1: A-B) Representative images of Ki67, ERα, PR and active-caspase 3 immunostaining of mammary glands from 5-week-old **(A)** and 3 to 6-month-old **(B)** virgin WT and C451A-ERα mice (t-test, **P* <0,05; scale bar=100 μm). The proportion of epithelial cells expressing Ki67 (both on distal ducts and terminal end buds (TEB), ERα, PR and active caspase 3 was quantified as percentage of total epithelial cells. On B) Confocal images of mammary gland epithelium after immunostaining with anti-K5(Green) and K8 (magenta) primary antibodies (scale bar=20μm or 5μm). **C)** Levels of the ERα protein normalized to GAPDH in mammary glands from 5-week-old or 3-6-month-old C451A-ERα and WT mice were assessed using Western blotting. Uteri were used as positive controls (C+) (two-way ANOVA, ns: not significant).

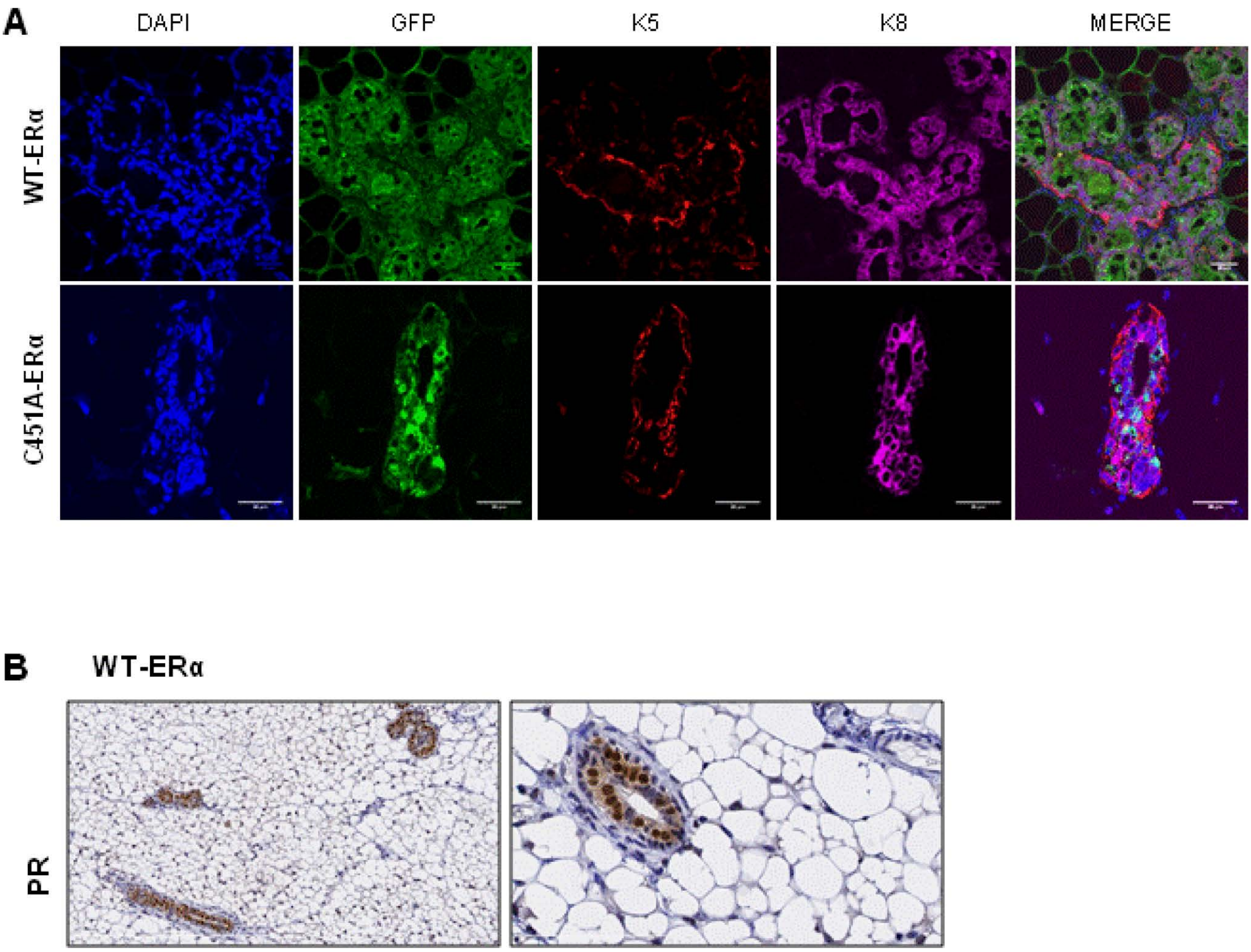

Figure S2: Immunostaining of pregnant 3-week-old WT mice mammary glands engrafted with a piece of mammary epithelium from C4514-ERα or WT mice

A) Confocal imaging of immunostainings using anti-GFP (green), -K5 (red) and -K8 (magenta) primary antibodies on mammary glands from 16 days pregnant mice grafted with ducts from WT or C451A-ERα mice (scale bar=26µm). **B)** Positive control of the PR staining from experiment in Figure 2C (scale bar=200 µm and 50µm)

Development • Supplementary information

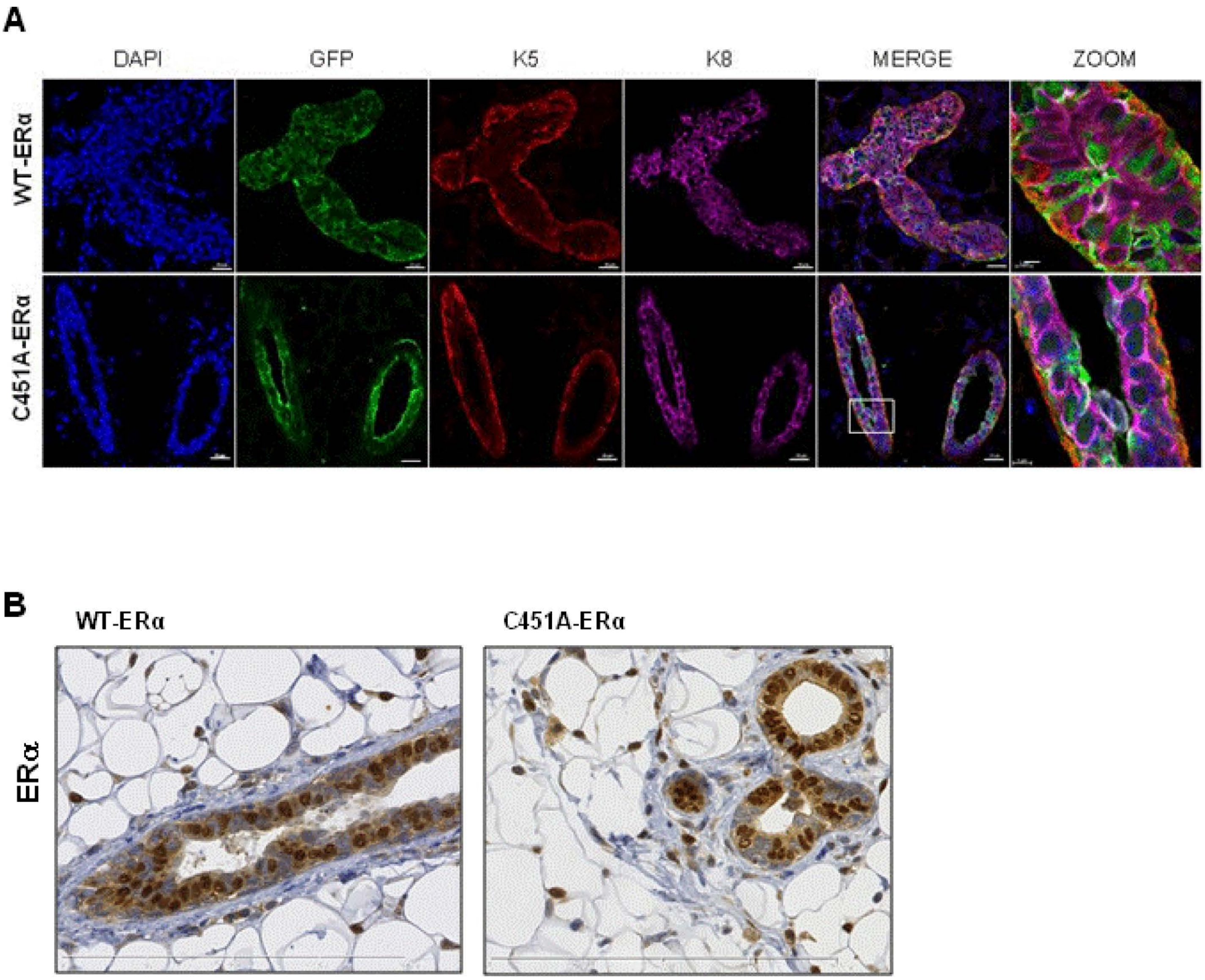

Figure S3: Immunostaining of the mammary epithelium 8 weeks after transplantation of basal cells from virgin C451A-ERα or WT mice

A) Confocal images after immunostaining using anti-GFP (green), -K5 (red), -K8 (magenta) and Dapi (blue). (Scale bar=20µm and in the high magnification image=5µm). **B)** Immunohistochemistry using anti-ERα (6F11) antibody. (scale bar=200 µm).

Development • Supplementary information

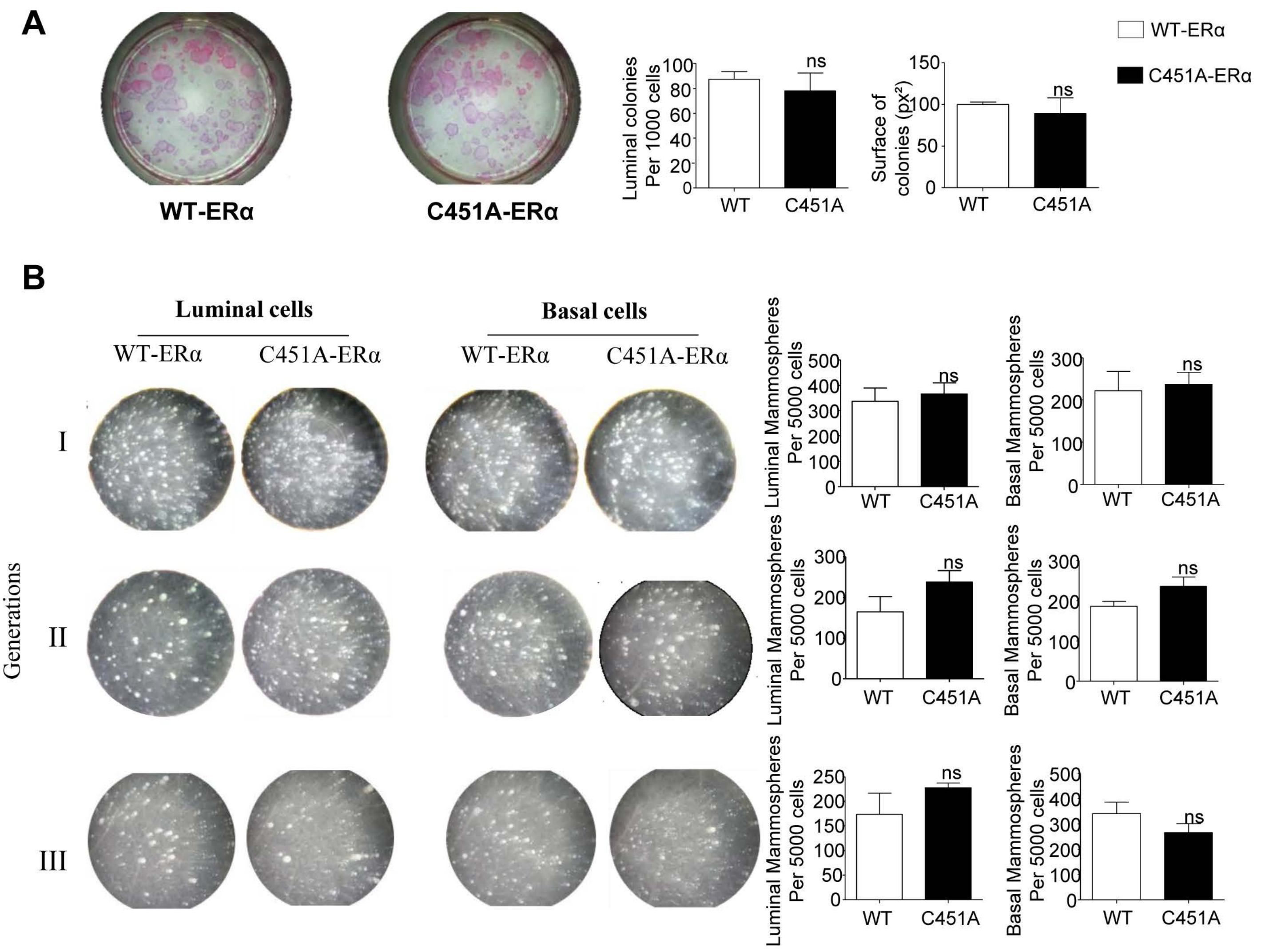

Figure S4: The C451A-ERα mutation does not modify the clonogenic capacity of luminal and basal cells in vitro, but impacts matrix degradation

A) Hematoxylin-eosin staining was performed to evaluate the clonogenic capacity of luminal $CD24^{+}CD29^{lo}$ cells seeded on irradiated fibroblast feeder cells after 7 days in culture. Box plots show the number of luminal colonies generated when 1000 cells were plated, and area of the different obtained clones (n= 3 independent experiments from luminal WT or ERα-C451A cells pooled from 2 to 4 mice (t-test, the P value was not significant)).

B) Representative images of mammosphere colony forming assays from luminal and basal cells grown on matrigel. After one generation, cells from the colonies were collected and plated again for the colony forming assay at generations II and III. The data indicate the number of mammospheres obtained from 5000 cells (n=4 independent experiments from luminal WT or C451A-ERα cells pooled from 2 to 4 mice each (t-test, the P value was not significant).

Development • Supplementary information

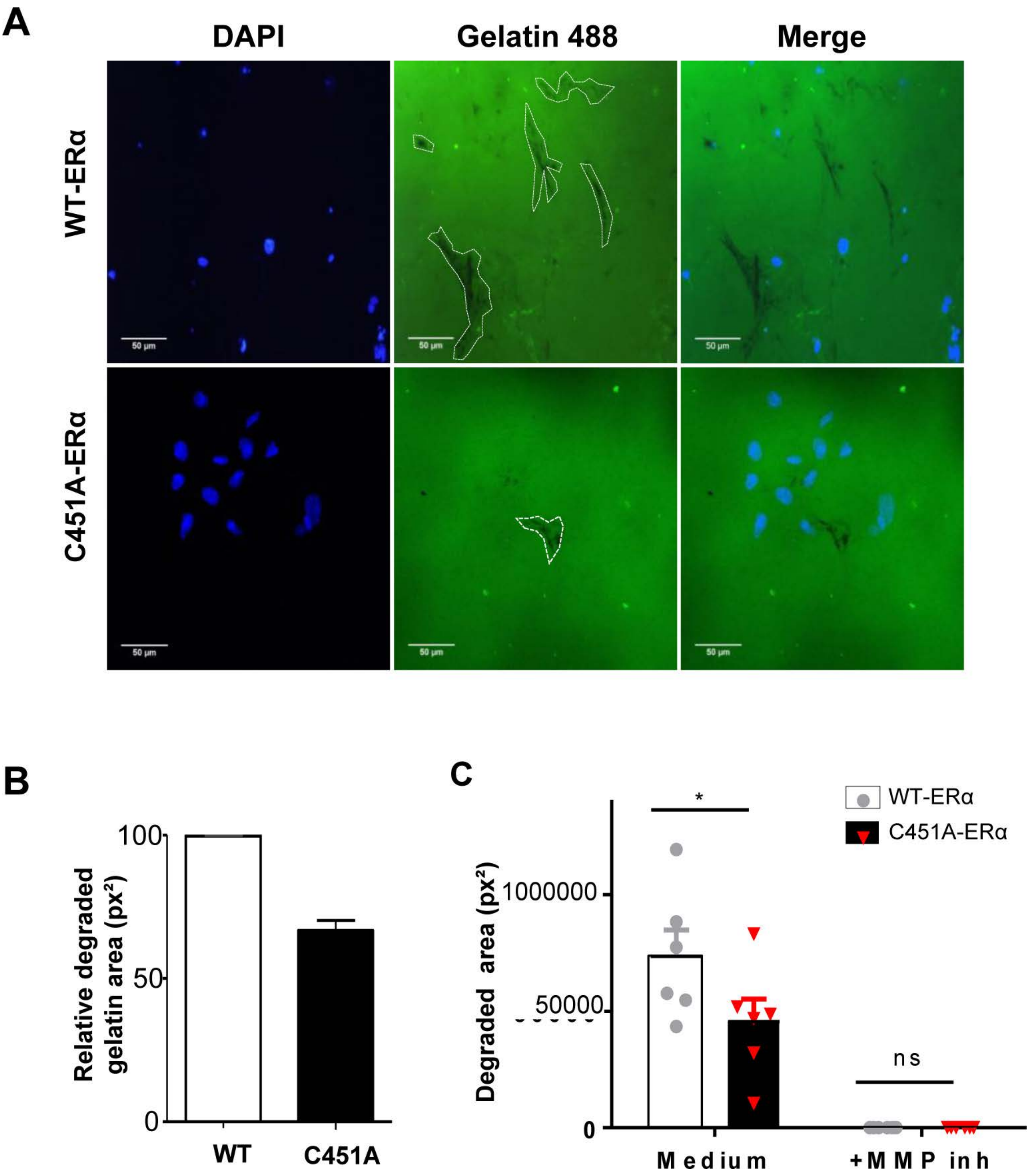

Figure S5: Images of immunofluorescence staining of green-fluorescent gelatin degradation by double-sorted CD29^{hi}CD24^{+} basal cells *in vitro*

A) Representative images of degraded A488-labeled gelatin appearing in black. The areas of degraded gelatin were measured in at least 18 fields per genotype in each experiment and an average of 281 cells was counted from each genotype in each experiment (scale bar=50µm). **B)** The area of gelatin degraded by gate-sorted C451A-ERα basal cells was compared to the area of gelatin degraded by cells purified from their WT littermates within the same experiment. The graph shows the means of four independent experiments using WT or C451A-ERα cells pooled from 2 to 4 mice each (t-test, **P value<0.01). **C)** Inhibition of gelatin degradation was observed when the non selective MMP inhibitor (marimastat) was added on the culture medium.

Development • Supplementary information

A **Co-transplantation of GFP-positive C45A basal cells with WT-ERα luminal cells**

	Ratio BCs /LCs	Number cells	Nb of outgrowths/ Total grafts	% outgrowth success
Experiment 1	4/1	2000 BCs/500 LCs	1/10	10%
	1/1	2000 BCs/2000 LCs	2/10	20%
Experiment 2	1/1	2000 BCs/2000 LCs	4/8	50%
Experiment 3	5/1	5000 BCs/ 1000 LCs	3/6	50 %
	5/1	2500 BCs/ 500 LCs	2/6	33%
	1/1	2500/2500	3/5	60%

B

Ratio BCs/LCs	Number cells	Number analyzed	GFP+ BCs only (Unipotency)	GFP+ BCs and LCs (Pluripotency)
1/1	2000 BCs/ 2000 LCs	2	1	1 high LCs
5/1	2500 BCs/500 LCs	2 *	1	
1/1	2500 BCs/ 2500 LCs	3	2	1 (few LCs)
5/1	5000 BCs/ 1000 LCs	3	1	2 (1 few+ 1 high LCS)
		Total	5/9	4/9
	* among 2 analyzed, ducts were only visible in 1 sample			

C

2500 MASC C451A + 2 500 Lum WT
(BCs + few LCs GFP positive)

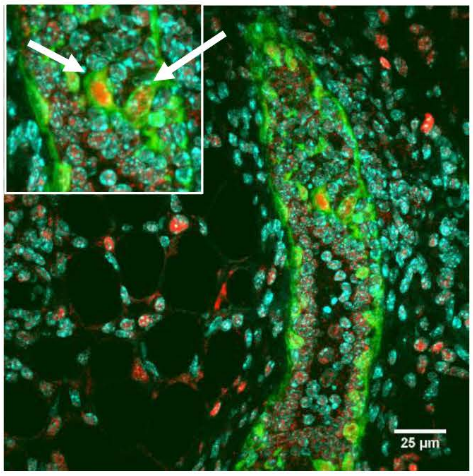

D

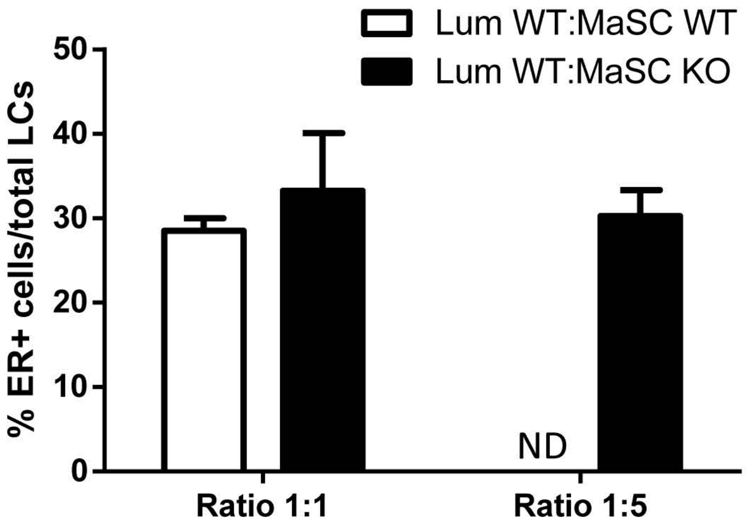

Figure S6: A) Recapitulative table of the co-transplantation experiments performed with different ratios of C451A GFP positive MaSCs with ERα-WT luminal cells and number of outgrowth success obtained for each conditions **B)** Number of grafts containing only GFP positive basal cells or giving bipotent GFP positive cells (i.e. both basal and luminal cells) obtained after co-transplantations of GFP-positive C451A-ERα basal cells with GFP-negative ERα-WT luminal cells, in the regenerated mammary gland 8 weeks after transplantation. Four of 9 mammary glands contained GFP-positive luminal cells, whereas when only GFP-negative luminal cells were injected while GFP-positive luminal cells were not detected in 5 out of 9. In grey, are underlined conditions given the higher proportion of success obtained. **C)** Confocal image of mammary gland epithelium after immunostaining using anti-GFP (Green), -ERa (MC20, red) and Dapi (Cyan) in epithelium 8 weeks after co-injection of GFP-positive C451A-ERα MASC mixed with GFP negative luminal cells from WT mice when MaSCs C451A gave rise to basal and few luminal GFP positive cells (right), with very rare GFP luminal cells were found ERα positive (white arrows). **D)** The proportion of ERα positive cells following immunohistochemistry using anti-ERa antibody was quantified in the regenerated mammary gland obtained 8 weeks after co-transplantations of GFP-positive C451A-ERα or WT basal cells with GFP-negative ERα-WT luminal cells.

Development • Supplementary information

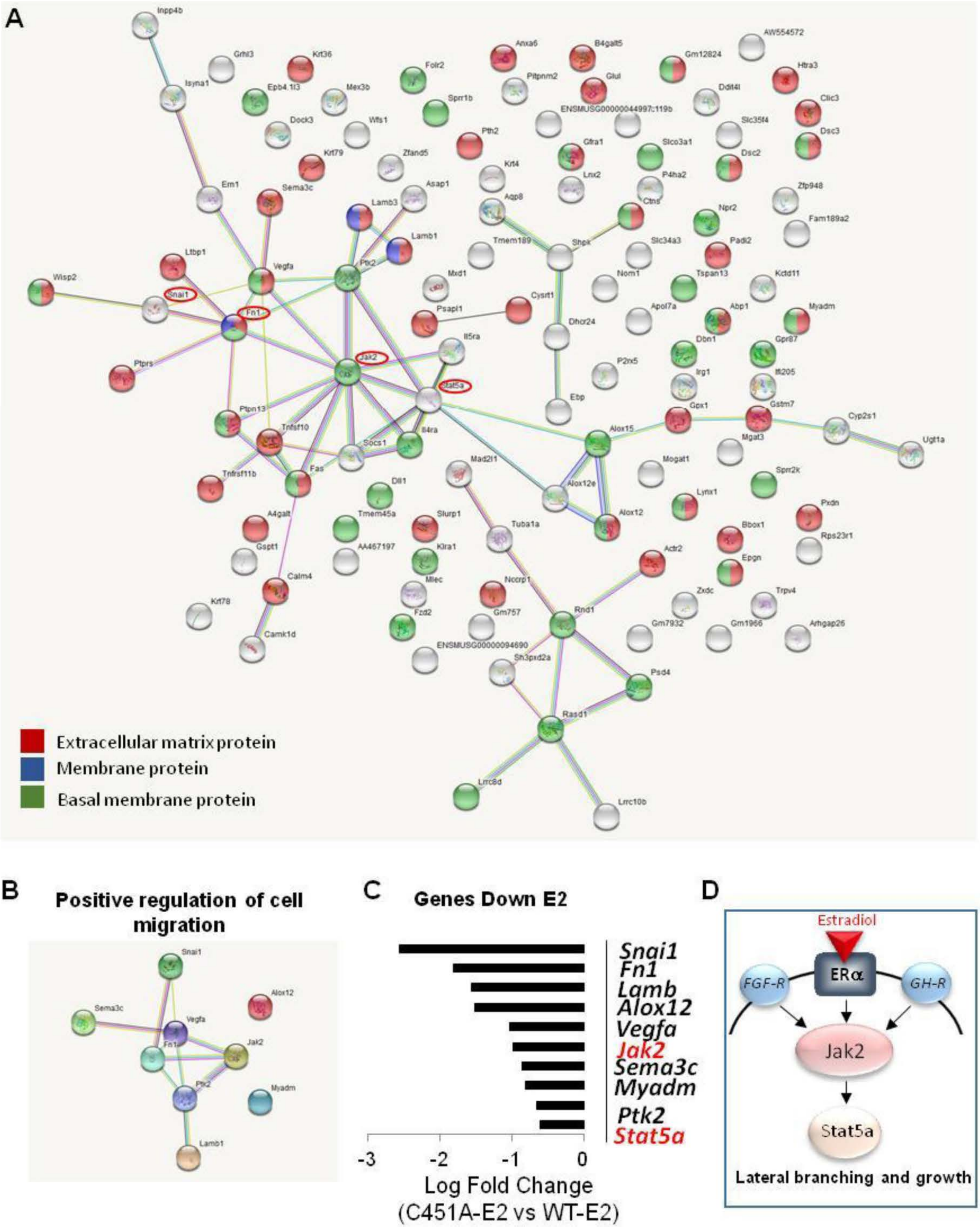

Development • Supplementary information

Figure S7: A) Predicted gene-gene interaction network (Search tool for the Retrievel of interacting Genes/StringV10) among genes significantly down-regulated in C451A versus WT mice by E2 (FC>1.5, P>0.05). Genes coloured in red belong to the GO category of «extracellular region», in blue to the «Plasma membrane» and in green to the « basal membrane protein». **B)** Gene network among the 9 genes found in the GO category of genes belonging to the positive regulation of cell migration, including *Stat5a*, a related gene with **C)** the corresponding Log fold change indicated in the right. **D)** Model of impact of membrane ERα on stem cells interfering with pathways induced by Growth-Hormone Receptors (GH-R) and FGF-R receptors impacting the Jak2/Stat5 signalling pathways, modified from Furth et al., 2011.

Development • Supplementary information

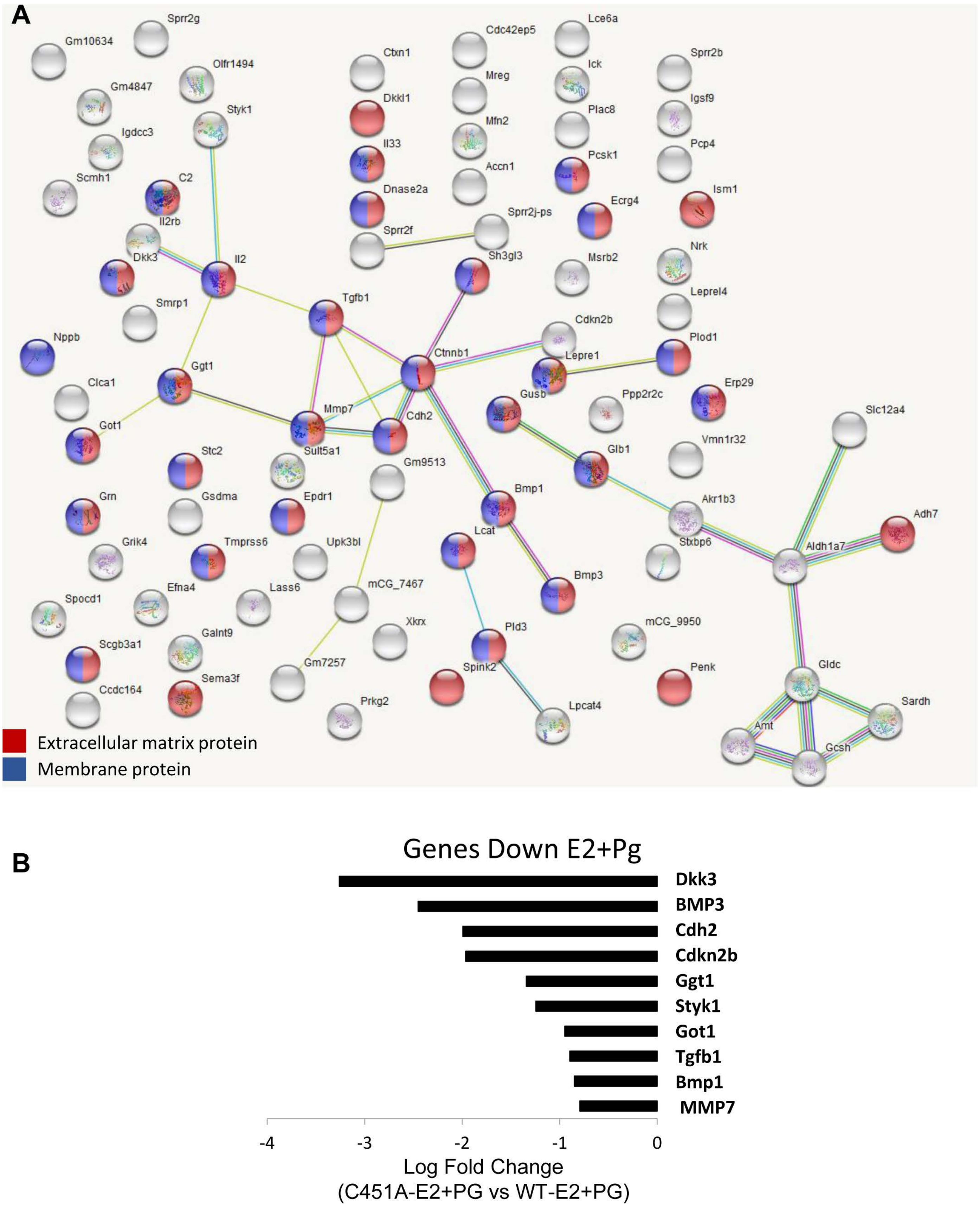

Figure S8: A) Predicted gene-gene interaction network (Search tool for the Retrievel of interacting Genes/StringV10) among genes significantly down-regulated in C451A versus WT mice by E2+Progesterone (Pg) (FC>1.5, P>0.05). Genes coloured in red belong to the GO category of «extracellular matrix protein», in blue to the «membrane protein». In **B)** the Log fold change of genes found in the network are indicated.

Development • Supplementary information

Table S1: **Summary of co-transplantation experiments performed mixing C451A-ERα MaSCs with either Sca1^{+} CD133+ or Sca1^{-} CD133^{-} WT luminal cells at a ratio of 5 basal/1 luminal cells.**

	Ratio BCs/LCs	Number cells	Mice grafted	Right	Left	Outgrowth right	Outgrowth left
**Experiment 1**	5/1	**5000/1000**	5	**MaSC C451A-ERα LCs WT Sca1^{+}**	**MaSC C451A-ERα LCs WT Sca1^{-}**	**1/5** (70% outgrowth- BCs GFP only)	0%
**Experiment 2**	5/1	**3000/600**	10	**MaSC C451A-ERα LCs WT Sca1^{+}**	**MaSC C451A-ERα LCs WT Sca1^{-}**	**1/10** (80% outgrowth- rare GFP LCs)	0%

Development • Supplementary information

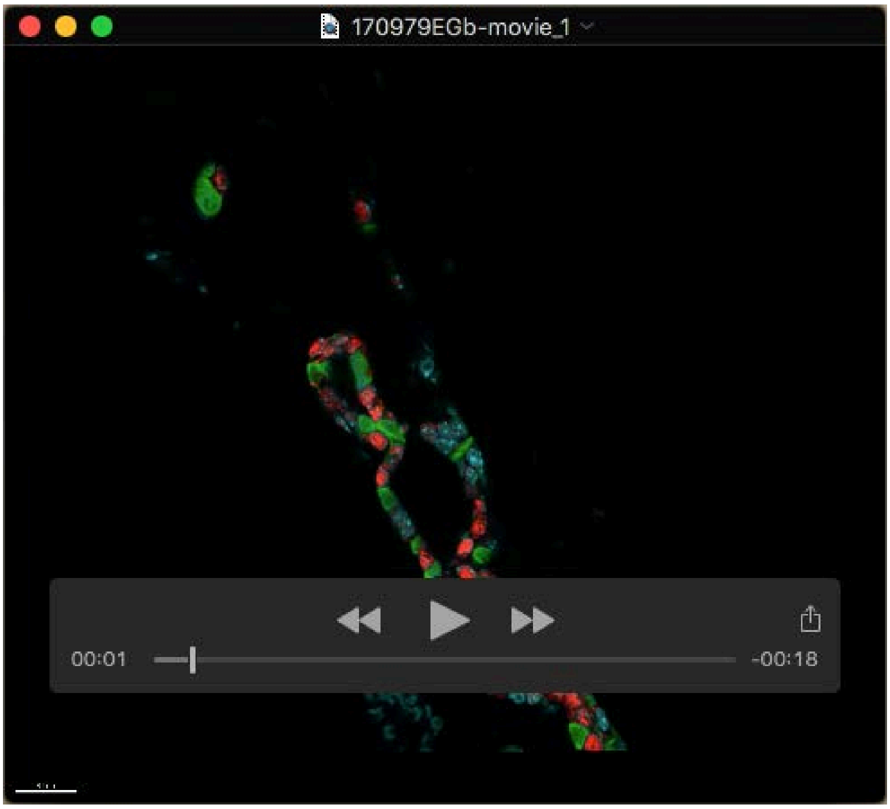

Movie 1: Movie showing different angles of the confocal images of the 3D-reconstructed outgrowths obtained 8 weeks after transplantation of a mixture of GFP positive C451A MaSCs with WT luminal cells, after labeling with anti-ERα (red) and anti-GFP (green) antibodies.
